# Diagnosis and management of invasive fungal infections due to non-*Aspergillus* moulds

**DOI:** 10.1093/jac/dkaf005

**Published:** 2025-03-14

**Authors:** C Orla Morrissey

**Affiliations:** Department of Infectious Diseases, Alfred Health, Melbourne, Victoria, Australia; Department of Infectious Diseases, School of Translational Medicine, Monash University, Melbourne, Victoria, Australia

## Abstract

Invasive fungal infection (IFI) due to moulds other than *Aspergillus* are a significant cause of morbidity and mortality. Non-*Aspergillus* mould (NAM) infections appear to be on the increase due to an ever-expanding population of immunocompromised hosts. In this review, Mucorales, *Scedosporium* species, *Lomentospora prolificans* and *Fusarium* species are examined in detail, and the microbiology, risk factors, diagnosis and treatment of emerging NAMs such as *Paecilomyces variotti*, *Purpureocillium lilacinum* and *Rasamsonia* are summarized. The challenges in diagnosis are emphasized and the emerging importance of molecular methods is discussed. Treatment of IFI due to NAMs is a multi-pronged and multi-disciplinary approach. Surgery, correction of underlying risk factors, and augmentation of the host immune response are as important as antifungal therapy. Many of these NAMs are intrinsically resistant to the currently licensed antifungal agents, so selection of therapy needs to be guided by susceptibility testing. There are new antifungal agents in development, and these have the potential to improve the efficacy and safety of antifungal treatment in the future. Ongoing research is required to fully delineate the epidemiology of NAM infections, and to develop better diagnostic tools and treatments so that outcomes from these infections can continue to improve.

## Introduction

Although *Aspergillus* is the major cause of invasive mould infections (70%–80%), non-*Aspergillus* moulds (NAMs) are more challenging to diagnose and treat and are associated with higher mortality rates.^1^ NAM infections appear to be increasing due to an expansion of the at-risk population, new immunotherapeutic agents available for cancer/haematological malignancy treatment, and selective pressures related to increasing use of broad-spectrum mould-active antifungal prophylaxis.^2–6^

In this review, the epidemiology and risk factors, diagnosis and treatment of NAMs, including Mucorales, *Scedosporium* spp., *Lomentospora prolificans* and *Fusarium* spp., will be examined. This review will provide clinicians with practical frameworks for the prompt diagnosis and treatment of NAMs in clinical practice and pay particular attention to cutting-edge diagnostic tools, novel antifungal agents and adjuvant therapies that are increasingly available for use in clinical practice.

## Methods

To provide clinicians with comprehensive guidance on how to diagnose and manage NAM infections a literature review of PubMed was undertaken up until 30 April 2024. The following terms were used: ‘adjunct’; ‘amphotericin B’; ‘antifungal’; ‘azole’; ‘basidiomycetes’; ‘blood culture’; ‘breakthrough’; ‘central nervous system’; ‘check point inhibitors’; ‘computed’; ‘corticosteroid’; ‘COVID-19’; ‘culture’; ‘cystic fibrosis’; ‘cytotoxic T-cells’; ‘deferasirox’; ‘diagnosis’; ‘disseminated’; ‘dissemination’; ‘duration’; ‘echinocandin’; ‘emerging’; ‘endophthalmitis’; ‘epidemiology’; ‘fosmanogepix’; ‘fungemia’; ‘fusariosis’; ‘*Fusarium*’; ‘G-CSF’; ‘GM-CSF’; ‘graft-versus-host-disease’; ‘granulocyte transfusion’; ‘hematological malignancy’; ‘hematopoietic stem cell transplant’; ‘histology’; ‘histopathological’; ‘histopathology’; ‘hyalohyphomycosis’; ‘hyperbaric oxygen’; ‘ibrexafungerp’; ‘identification’; ‘imaging’; ‘immunocompromise’; ‘immunosuppression’; ‘invasive fungal disease’; ‘invasive fungal infection’; ‘leukemia’; ‘*Lomentospora prolificans*’; ‘lymphoma’; ‘MALDITOF’; ‘microscopy’; ‘MIC’; ‘mold’; ‘molecular’; ‘mortality’: ‘MRI’; ‘Mucorales’; ‘mucormycosis’; ‘myeloma’; ‘nasal’; ‘neutropenia’; ‘non-*Aspergillus* mold’; ‘nose’; ‘olorofim’; ‘*Paecilomyces*’; ‘PCR’; ‘*Penicillium*’; ‘phaeohyphomycosis’; ‘probable’; ‘proven’; ‘*Pseudallescheria*’; ‘pulmonary’; ‘*Purpureocillium*’; ‘*Rasamsonia*’; ‘response’; ‘rhino-orbital-cerebral’; ‘risk factor’; ‘salvage’; ‘SARS-CoV-2’; ‘scedosporiosis’; ‘*Scedosporium*’; ‘*Schizophyllum commune*’; ‘*Scopulariopsis*’; ‘sequencing’; ‘serology’; ‘sinus’; ‘skin’; ‘solid organs transplant’; ‘species’; ‘surgery’; ‘susceptibility testing’; ‘suspected’; ‘*Talaromyces*’; ‘terbinafine’; ‘therapeutic drug monitoring’, ‘therapy’; ‘treatment’; ‘tyrosine kinase inhibitor’; ‘VT-1161’; ‘VT 1598’; ‘zygomycetes’; ‘zygomycosis’; ‘Zygomycota’.

## Mucorales

With the advent of molecular tools, a change in nomenclature has occurred, such that the term Zygomycota is now obsolete. All infections due to the order Mucorales are now termed mucormycosis and not zygomycosis.^7^ The most common members are *Rhizopus* spp., *Mucor* spp. and *Lichtheimia* spp. (formerly *Absidia* and *Mycocladus*). Other important members include *Rhizomucor*, *Cunninghamella*, *Apophysomyces* and *Saksenaea* spp. There are geographical variations in the relative frequency, with *Apophysomyces* most common in India but very rare in Europe.^8–13^

### Risk factors

Risk factors that should prompt a clinician to suspect mucormycosis include advanced haematological malignancy, poor performance status, allogeneic HSCT, solid organ transplant (SOT), poorly controlled diabetes mellitus (DM) and/or diabetic ketoacidosis (DKA), iron overload and deferoxamine therapy, prolonged corticosteroid use, burns and major trauma, and prior prophylaxis with azoles and echinocandins.^2,14–17^ More recently, Bruton tyrosine kinase inhibitors and severe acute respiratory syndrome coronavirus 2 (SARS-CoV-2) have been implicated as risk factors.^15,18–20^ Despite this, many patients with invasive mucormycosis have no identifiable risk factor. Again, geographical variations occur, with DM being the most common risk factor in India, and a haematological malignancy the most common in Europe and the USA.^17^

### Clinical manifestations

The most common clinical manifestation of mucormycosis is rhino-orbital-cerebral (ROC) infection (proptosis, ophthalmoplegia, nasal drip/epistaxis, hemiplegia,) especially in those with poorly controlled DM and/or DKA.^16^ The lungs are the second most common site of mucormycosis. The most common risk factor for pulmonary mucormycosis (PM) is a haematological malignancy or HSCT, and patients commonly present with fever, pleuritic chest pain, persistent cough and haemoptysis.^9,10,21^ Other sites of infection include the skin, especially after major trauma, but they can also be seen in disseminated disease.^22^ Lesions are commonly painful, erythematous, papular/nodular with some having a central necrotic area. Gastrointestinal disease can occur in malnourished patients and in low-birthweight infants as well as in the immunocompromised (e.g. haematology patients, SOT). Symptoms include abdominal pain, diarrhoea, gastrointestinal bleeding, abdominal distension and perforation.^23^

### Diagnosis

**Figure 1.**
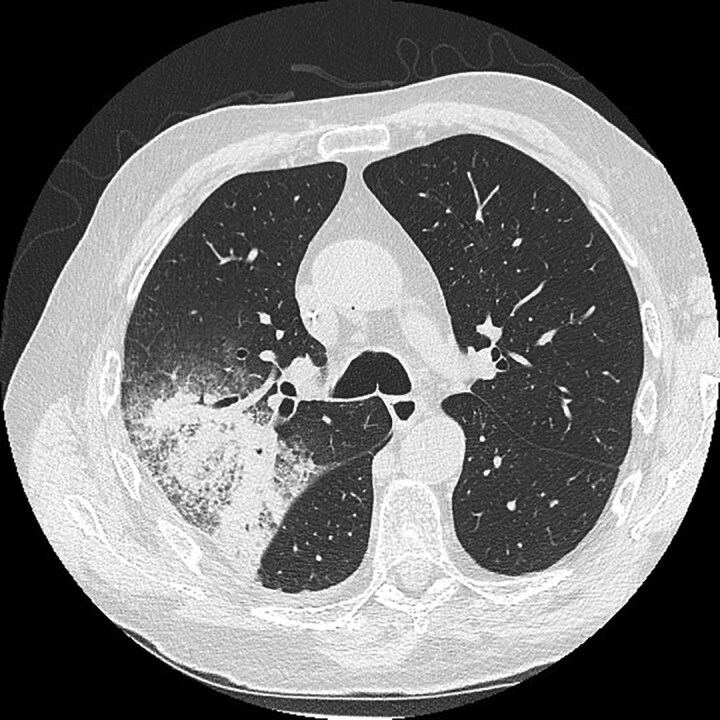
CT scan image (axial view) of a reverse halo sign. This is defined as a central area of ground-glass opacification surrounded by a denser peripheral area of consolidation. Courtesy of Professor Samantha Ellis, Department of Radiology, Alfred Health, Melbourne.

Diagnosis is difficult and challenging as conventional microbiological tests lack sensitivity and no specific biomarkers have been validated. As disseminated disease can occur in 16% to 40%, blood cultures should be taken, and a detailed examination of the skin should be performed to detect any suspicious lesions for biopsy.^24–27^ A chest CT scan should be performed in all cases of suspected PM. Signs that increase the likelihood of PM over invasive pulmonary aspergillosis include the reverse-halo sign (Figure 1), vessel-occlusion sign (contrast flow interruption), >10 nodules and a pleural effusion (Table 1).^28–30^ A sinus CT is recommended in all cases of suspected ROC mucormycosis and when PM is diagnosed to determine the extent of infection. Sinus CT scan findings consistent with ROC mucormycosis include mucosal thickening, sinus opacification, bony erosions (which may be late) and extension into the neighbouring tissues.^67^ With any extension an MRI scan should also be performed to better delineate the full extent of the infection in the brain and eyes. Imaging is also very important to identify appropriate sites for biopsy. Table 1 details the recommended tests to be used to diagnose mucormycosis.

**Table 1.** Diagnosis of invasive fungal infection due to Mucorales, *Scedosporium* species, *Lomentospora prolificans* and *Fusarium* species

Organism	Diagnostic test	Specifics of diagnostic test	Comments	Relevant references
Mucorales	Blood cultures		Should be performed in every patient with suspected mucormycosis	^24–27^
	Imaging	CT scans of sinuses, lungs and abdomen MRI scans of brain, eyes, spine, bones and joints	Imaging should be performed to determine the sites and extent of infection, detect any suitable sites for biopsy and assess responses to treatment Features characteristic of PM include vessel-occlusion sign (contrast flow interruption), >10 nodules and a pleural effusion (Figure 1)	^28–30^
	Microscopy^a^	To identify genus and species	Non-pigmented; wide, ribbon-like with irregular branching; 6–16 μm in width; non-septate or pauci-septate hyphae (Figure 2)	^31^
	Culture^a^	To identify genus and species and for antifungal susceptibility testing	Avoid homogenization of tissue and incubate at 30°C and 37°C to increase yield. Depends on species but colonies are usually cottony and white to grey/brown in colour	^31^
	MALDI-TOF MS	Used as an adjunct for species identification	Use of validated in-house databases associated with better accuracy. Only 86% of isolates correctly identified using commercially available database in one multi-centre study	^32^
	Serology	GM and 1,3-β-D-glucan assays	Not recommended to use as an adjunct for the diagnosis of mucormycosis. GM is not present in the cell wall and the amount of 1,3-β-D-glucan present is below the lower limit of detection of the assay	
	Molecular	Adjunct for the diagnosis of mucormycosis	In-house using a variety of DNA targets (ITS, 18S rDNA, 28S rDNA) and techniques (e.g. PCR ± sequencing) Fresh tissue is preferred as sensitivity is greater than for FFPE tissue Can be used on blood, CSF and BAL fluid	^33^
		Mucorales-specific assays for use in serum	Sensitivity of 81% for proven or probable mucormycosis in a retrospective study of 44 patients Adjunct for the diagnosis of mucormycosis, if available	^34,35^
	Susceptibility testing	CLSI EUCAST	Should be performed on all isolates. No interpretative breakpoints. MIC values used to guide antifungal therapy	^36–38^
*Scedosporium* spp.	Blood cultures		Should be performed in every patient with suspected scedosporiosis	
	Imaging	CT scans of sinuses, lungs and abdomen MRI scans of brain, eyes, spine, bones and joints	Imaging should be performed to determine the sites and extent of infection, detect any suitable sites for biopsy and assess responses to treatment No specific features to differentiate it from other NAMs	
	Microscopy^a^	To identify genus and species	Septate hyphae that branch at 60–70°. Conidiogenous cells are usually flask-shaped. Conidia are usually unicellular and oval-shaped. Presence of ascocarp and/or pyriform adventitious conidia helps in differentiation from other NAMs (Figure 3)	
	Culture^a^	To identify genus and species and for antifungal susceptibility testing	Grow rapidly. Darker on the reverse side (dark grey to black) as compared with the upper surface (grey to white). Mycelial tufts are fine and short. Cultures tend to become cottony with maturity (Figure 4)	
	MALDI-TOF MS	Used as an adjunct for species identification	Usually only successful if commercial databases supplemented with in-house libraries	
	Serology	GM	Not recommended to use as an adjunct for the diagnosis of scedosporiosis	^39^
		1,3-β-D-glucan assays	Can be used as an adjunct for the diagnosis of fusariosis	
	Molecular	Adjunct for diagnosis and identification to species level	No standardized assays available. Any assays have only been well studied on CF patients. Recommend pan-fungal PCR for use on tissue specimens and ITS1, ITS2 and β-tubulin and calmodulin sequencing for use on cultures	^40–42^
	Susceptibility testing	CLSI EUCAST	Should be performed on all isolates. No interpretative breakpoints. Similar results obtained with Sensititre YeastOne Y010 assay as with CLSI and EUCAST. MIC values used to guide antifungal therapy. Generally high MIC values of amphotericin B, isavuconazole and itraconazole. Lowest MIC values seen with voriconazole followed by posaconazole. Echinocandins can exhibit activity against some isolates	^37,38,43,44^
*Lomentospora prolificans*	Blood cultures		Should be performed in every patient with suspected invasive infection due to *L. prolificans* (positive in up to 72%)	^45^
	Imaging	CT scans of sinuses, lungs and abdomen MRI scans of brain, eyes, spine, bones and joints	Imaging should be performed to determine the sites and extent of infection, detect any suitable sites for biopsy and assess responses to treatment No specific features to differentiate it from other NAMs	
	Microscopy^a^	To identify genus and species	Pigmented, flask-shaped conidiophores that are swollen at the bases (Figure 5)	
	Culture^a^	To identify genus and species and for antifungal susceptibility testing	Characterized by rapid growth. Colonies are olive to black in colour and the surface is suede to downy	
	MALDI-TOF MS	Used as an adjunct for species identification	Usually only successful if commercial databases supplemented with in-house libraries	
	Serology	GM	Not recommended to use as an adjunct for the diagnosis of invasive infection due to *L. prolificans*	
		1,3-β-D-glucan assays	Can be used as an adjunct for the diagnosis of fusariosis	
	Molecular	Adjunct for diagnosis and identification to species level	Several non-standardized assays available (oligoarray, multiplex PCR, pan-fungal PCR and sequencing, and PCR and reverse line blot hybridization A multiplex fungal or pan-fungal PCR assay followed by sequencing, hybridization or microarray is recommended	^41,46–53^
	Susceptibility testing.	CLSI EUCAST	Mostly resistant to all currently licensed antifungal agents. Some isolates have low MIC values against voriconazole and posaconazole (but less so)	^37,38,43,44^
*Fusarium* spp.	Blood cultures		Should be performed in every patient with suspected fusariosis	^54^
	Imaging	CT scans of sinuses, lungs and abdomen MRI scans of brain, eyes, spine, bones and joints	Imaging should be performed to determine the sites and extent of infection, detect any suitable sites for biopsy and assess responses to treatment Less likely to demonstrate halo-sign(s) than invasive *Aspergillus* infection	^55,56^
	Microscopy^a^	To identify genus and species	Hyaline hyphae with pigmented banana- or canoe-shaped multicellular macroconidia with a foot cell at the base (Figure 7). Adventitious sporulation can help in differentiation from *Aspergillus*	
	Culture^a^	To identify genus and species and for susceptibility testing	Usually, colonies are pink or violet in the centre with a lighter periphery. *F. solani* SC differ as conidia are usually blue-green	
	MALDI-TOF MS	Used as an adjunct for species identification	Only available in research centres for *Fusarium* spp.	^57^
	Serology	GM and 1,3-β-D-glucan assays	Are recommended for use as an adjunct for the diagnosis of fusariosis. GM assay cross-reacts with *Fusarium* spp. In one study GM assay had a sensitivity of 83%, a specificity of 67% and in 73% was positive before clinical signs and symptoms developed. Prognostic value as well as persistently positive results correlate with a poor outcome When the threshold for the 1,3-β-D-glucan assay is >80 pg/mL and two sequential positive results are used to diagnose invasive fusariosis, the sensitivity is high (90%) but the specificity is low at 60%	^58^ ^59^
	Molecular	Adjunct for diagnosis and identification to species level	Several methods available: AFLP, loop-mediated isothermal amplification, MLST and RT-PCR Sequencing of the *TEF1-α* gene can differentiate between the different species: *F. oxysporum*, *F. solani*, *F. keratoplasticum*, *F. petroliphilum*, *F. napiforme*, *F. falciforme*, *F. pseudensiforme* and *F. dimerum* The use of *TEF1-α* gene sequencing with multiplex PCR or DNA microarray hybridization can detect *F. solani* and *F. oxysporum* in neutropenic patients The use of *TEF1-α* gene sequencing with pan-fungal PCR (ITS target) and Luminex multi-analyte profiling technology can detect *F. solani*, *F. oxysporum*, *F. verticillioides*, and *F. proliferatum* (considered a pan-*Fusarium* PCR assay) Can be used as in blood, CSF and tissue Needs further validation before it is used routinely in clinical practice Recommend *TEF1-α* gene sequencing as an adjunct for diagnosis and speciation, if available RPB2 can be recommended for use in species identification	^41,48,50,51,60–64^
	Susceptibility testing	CLSI EUCAST	Usually have high MIC values to most of the currently licensed antifungal agents. However, the MIC values vary from species to species hence the importance of species identification Amphotericin B and voriconazole usually have activity against most species, except for *F. solani* SC and *F*. *verticillioides* (resistant to voriconazole and high MIC values of amphotericin B) *Fusarium* spp. are intrinsically resistant to echinocandins Correlation of clinical outcome to MIC values is unclear as data are conflicting	^37,38,65,66^

AFLP, amplified fragment length polymorphism; BAL, bronchoalveolar lavage fluid; CF, cystic fibrosis; FFPE, formalin-fixed paraffin-embedded; GM, galactomannan; ITS, internal transcriber sequence; NAM, non-*Aspergillus* moulds; PM, pulmonary mucormycosis; rDNA, ribosomal DNA; RPB2, RNA polymerase II second largest subunit; RT-PCR, real-time PCR; SC, species complex.

^a^A detailed description of the microscopy and culture features of each NAM can be found at Mycology Online (http://www.mycology.adelaide.edu.au).

**Figure 2.**
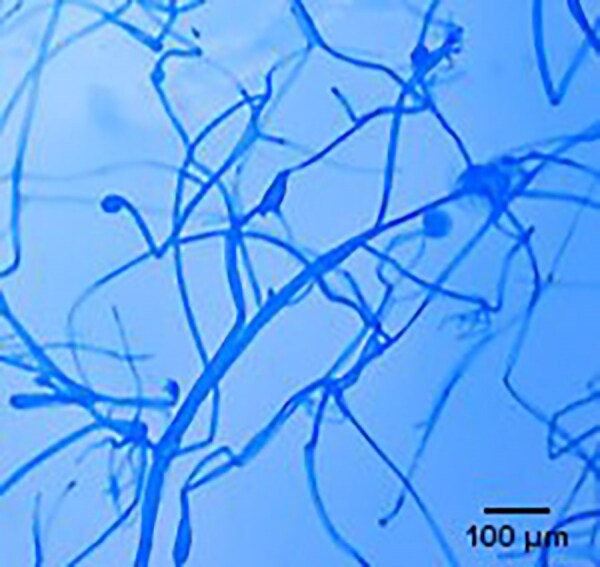
*Rhizopus microsporus* showing dispersed hyphae/stolon structure. Low objective view. Prepared using adhesive tape method and stained with lactophenol blue. Courtesy of Professor Wieland Meyer, Westerdijk Fungal Biodiversity Institute and Ms Krystyna Maszewska, Dr Catriona Halliday, Mr Alex Khan, Mr Tsung-Yu Pai and Ms Georgia Clementine Wunderlich, Centre for Infectious Diseases and Microbiology, Westmead Hospital, Westmead, NSW, Australia.

It is critically important to obtain specimens for microscopy, culture, and histopathological and/or molecular testing when mucormycosis is suspected. The type of specimen is dependent on the site of infection. For example, in suspected PM a bronchoalveolar lavage may provide the prerequisite specimens. As Mucorales are wide and ribbon-like in structure with irregular branching and non-septate or pauci-septate hyphae (Figure 2), they only grow 15%–25% of the time.^31^ As a result, more invasive biopsies (CT-guided, open lung) may be needed (Table 1). In this setting, it is important that clinicians liaise with the microbiology laboratory to ensure that these specimens are not minced/homogenized. This will improve the chances of a positive culture. A culture is very important for identification to species level and antifungal susceptibility testing (Table 1). Commercially available monoclonal antibodies against Mucorales may be used on histological specimens to differentiate between Mucorales and *Aspergillus*.^68^ Definitive identification is made by sequencing [internal transcriber spacer (ITS)] and MALDI-TOF MS. If histology shows features consistent with Mucorales but culture is negative, then PCR testing should be performed on the tissue specimen. Several PCR assays have been developed for the detection of Mucorales in serum and blood, with sensitivities and specificities of 75%–100% and 84.6%–100%, respectively. Some of these assays are in-house, but commercial assays are also available (Mucorgenius^®^, PathoNostics). Comprehensive standardization and validation are still required. A full description of these PCR assays for Mucorales is beyond the scope of this review, but Lamoth and Kontoyiannis^33^ provide an excellent overview of the topic. If a PCR assay is readily available, then clinicians should use them as an adjunct for diagnosis. As galactomannan (GM) is not a component of Mucorales and the amounts of 1,3-β-D-glucan produced by members of this order are below the lower limit of detection, these two serological assays are not recommended for the diagnosis of mucormycosis (Table 1).

### Treatment

There are four key elements to the treatment of mucormycosis. These include prompt directed antifungal therapy (optimized using therapeutic drug monitoring),^69–71^ surgical debridement, adjuvant therapies and reversal of the underlying risk factors (e.g. treat DKA and obtain good control of DM). A delay in antifungal therapy beyond 3 days increases mortality from 33% to 72%.^21^ Liposomal amphotericin B (L-AMB) is still considered as the drug of choice for treatment of mucormycosis at a dose of 5 mg/kg IV daily unless there is CNS involvement, whereupon a dose of 10 mg/kg IV is recommended (Table 2).^72,73^ Posaconazole as modified-release tablet or IV formulations, and isavuconazole orally or IV are alternatives as first-line (if significant baseline renal impairment) or salvage therapy (if failed primary therapy with L-AMB).^75,106,107^ Table 2 provides details on the dosing of the different antifungal agents and the evidence base for the recommendations for treatment in different clinical scenarios. Combination therapy is not routinely recommended as first-line treatment; however, L-AMB and an echinocandin (likely a class effect) may be used in selected critically ill or severe cases (Table 2).^78–80^ A switch from IV to oral therapy is usually recommended once there has been a stable or partial response to treatment. The liquid suspension formulation of posaconazole is not recommended for treatment unless none of the other recommended commercially available antifungal agents are available (Table 2).^108^ Of all the novel antifungal agents that are in development (Table 3), fosmanogepix (targets glycosylphosphatidylinositol-anchored protein maturation by inhibiting the fungal enzyme Gwt1) appears to be the only one with *in vitro* activity against some organisms of the Mucorales order [minimum effective concentration (MEC) values of ≤1 mg/L against some isolates, but most have MEC values of 4–16 mg/L (Table 3)].^109,110,122,123^ A Phase II trial has just been terminated to make way for a Phase III trial for the same indication (i.e. examining the efficacy of fosmanogepix in the treatment of *Aspergillus* or rare moulds) (Table 3).

**Table 2.** Treatment of invasive fungal infection due to Mucorales, *Scedosporium* species, *Lomentospora prolificans* and *Fusarium* species

Organism	Antifungal regimen		Formulations, dosing, route	Comments	Relevant references
Mucorales	First-line: Recommended Alternative(s) Salvage: Recommended Alternative(s).	L-AMB Posaconazole^a^ Isavuconazole^a,b^ ABLC L-AMB plus caspofungin Posaconazole^a^ Isavuconazole^a,b^ L-AMB ABLC L-AMB plus caspofungin L-AMB plus posaconazole^a^ Posaconazole suspension^a^	5 mg/kg IV daily MR tablets/IV; 300 mg twice daily for two doses then 300 mg once daily thereafter 200 mg PO/IV three times a day for 2 days then 200 mg PO/IV daily thereafter 5 mg/kg IV daily L-AMB: 5 mg/kg IV daily Caspofungin: 70 mg IV daily on Day 1 then 50 mg IV daily thereafter MR tablets/IV; 300 mg twice daily for 2 doses then 300 mg once daily thereafter 200 mg PO/IV three times a day for 2 days then 200 mg PO/IV daily 5 mg/kg IV daily 5 mg/kg IV daily L-AMB: 5 mg/kg IV daily Caspofungin 70 mg IV daily on Day 1 then 50 mg IV daily thereafter L-AMB: 5 mg/kg IV daily Posaconazole: MR tablets/IV; 300 mg twice daily for two doses then 300 mg once daily thereafter Posaconazole suspension: 400 mg PO twice daily 400 mg PO twice daily	10 mg/kg IV daily if CNS disease If baseline renal impairment MoveOn Study compared first-line posaconazole (MR tablets/IV) with L-AMB + posaconazole (MR tablets/IV) with L-AMB alone Day 42 favourable response: 80% (4/5) vs 27.8% (5/18) vs 20% (3/15), respectively If baseline renal impairment VITAL trial: proven or probable mucormycosis cases treated with first-line isavuconazole c/w with external controls from the FungiScope Registry EOT favourable responses 32% (6/19) in cases Day 42 crude all-cause mortality 33% vs 39% (weighted all-cause mortality 33% vs 41%; *P* = 0.595) Not recommended in CNS disease L-AMB 10 mg/kg IV daily if CNS disease Consider continuing with 70 mg IV daily of caspofungin beyond Day 1 in critically ill patients and in those who weigh >80 kg Can use any echinocandin as likely a class effect Significantly greater treatment success of ROCM with the combination c/w polyene monotherapy (100% vs 45%; *P* = 0.02) Combination recommended only if severe disease or in a critically ill patient Where primary treatment with L-AMB has failed Where primary treatment with L-AMB has failed 10 mg/kg IV daily if CNS disease If primary therapy with posaconazole or isavuconazole has failed If primary therapy with posaconazole or isavuconazole has failed Not to be used in cases with CNS disease L-AMB 10 mg/kg IV daily if CNS disease Consider continuing with 70 mg IV daily of caspofungin beyond Day 1 in critically ill patients and in those who weigh >80 kg Can use any echinocandin as likely a class effect Combination recommended only if severe disease or in a critically ill patient L-AMB 10 mg/kg IV daily if CNS disease Any formulation of posaconazole can be used but prefer MR tablets/IV if available Cases of proven or probable mucormycosis identified from two registries (SEIFEM and FungiScope): 56% (18) treated with combination as salvage therapy had a favourable response Only if limited availability of other antifungal agents	^72–74^ ^75^ ^76^ ^77^ ^78–80^ ^75^ ^76^ ^77^ ^78–80^ ^81^
*Scedosporium* spp.	First-line:Recommended Alternative(s) Salvage:Recommended Alternative(s)	Voriconazole^a^ Posaconazole^a^ Posaconazole^a^ plus terbinafine Voriconazole^a^ Addition of an echinocandin and GM-CSF to pre-existing treatment with voriconazole^a^ Posaconazole^a^	6 mg/kg PO/IV twice daily for two doses then 4 mg/kg PO/IV twice daily thereafter MR tablets/IV; 300 mg twice daily for two doses then 300 mg once daily thereafter Posaconazole: MR tablets/IV; 300 mg twice daily for two doses then 300 mg once daily thereafter Terbinafine: 250 mg PO twice daily 6 mg/kg PO/IV twice daily for two doses then 4 mg/kg PO/IV twice daily thereafter Caspofungin 70 mg IV daily on Day 1 then 50 mg IV daily thereafter Or Anidulafungin 200 mg IV daily on Day 1 then 100 mg IV daily thereafter Or Micafungin 100 mg IV daily MR tablets/IV; 300 mg twice daily for two doses then 300 mg once thereafter	IV treatment is recommended initially. Step-down to oral therapy once clinically stable When MIC values of posaconazole are high, extrapolating from the treatment of *L. prolificans* Failed or intolerant of L-AMB or posaconazole given as primary treatment Consider continuing with 70 mg IV daily of caspofungin beyond Day 1 in critically ill patients and in those who weigh >80 kg Consider increasing the dose by 50%–75% beyond Day 1 in critically ill patients Consider increasing all doses by 50% in obese patients Consider using 150 mg daily in critically ill patients Seek expert advice from a haematologist regarding dosage of GM-CSF	^82,83^ ^46,83–87^ ^88^ ^89^ ^90^
*Lomentospora prolificans*	First-line:Recommended Alternative(s) Salvage:Recommended	Voriconazole^a^ and terbinafine Voriconazole^a^ plus L-AMB Voriconazole^a^ plus micafungin Voriconazole^a^ Voriconazole^a^-based combination regimen	Voriconazole: 6 mg/kg PO/IV twice daily for two doses then 4 mg/kg PO/IV twice daily thereafter Terbinafine: 250 mg PO twice daily Voriconazole: 6 mg/kg PO/IV twice daily for two doses then 4 mg/kg PO/IV twice daily thereafter L-AMB: 5 mg/kg IV daily Voriconazole: 6 mg/kg PO/IV twice daily for two doses then 4 mg/kg PO/IV twice daily thereafter Micafungin: 100 mg IV daily 6 mg/kg PO/IV twice daily for two doses then 4 mg/kg PO/IV twice daily thereafter Voriconazole: 6 mg/kg PO/IV twice daily for two doses then 4 mg/kg PO/IV twice daily thereafter	Step-down to oral therapy once clinically stable 45% (8/18) were alive at Day 42 and the probability of survival was significantly greater in those receiving the combination Only a small number of cases treated with this combination 10 mg/kg IV daily if CNS disease Only a small number of cases treated with this combination Consider using 150 mg daily in critically ill patients As monotherapy if terbinafine is not available The remainder of the regimen is dependent on the response to, or intolerance of other prior antifungal agents used. Seek expert advice	^83–87,91,92^ ^83,87,91^ ^83,87,91^ ^82,91,93,94^
*Fusarium* spp.	First-line: Recommended Alternative(s) Salvage: Recommended Alternative(s)	Voriconazole^a^/L-AMB/ABLC Voriconazole^a^ plus L-AMB/ABLC Voriconazole^a^ Voriconazole^a^-based combination regimen Posaconazole^a^ L-AMB or ABLC	Voriconazole: 6 mg/kg PO/IV twice daily for two doses then 4 mg/kg PO/IV twice daily thereafter L-AMB/ABLC: 5 mg/kg IV daily Voriconazole: 6 mg/kg PO/IV twice daily for two doses then 4 mg/kg PO/IV twice daily thereafter L-AMB/ABLC: 5 mg/kg IV daily Voriconazole: 6 mg/kg PO/IV twice daily for two doses then 4 mg/kg PO/IV twice daily thereafter Voriconazole: 6 mg/kg PO/IV twice daily for two doses then 4 mg/kg PO/IV twice daily thereafter Posaconazole: MR tablets/IV; 300 mg twice daily for two doses then 300 mg once thereafter 5 mg/kg IV daily	Voriconazole or lipid formulations of amphotericin B cannot be recommended one over the other IV voriconazole is recommended initially with step-down to oral once clinically stable Global study of 236 patients: 90 day probability of survival was 53% in those treated with voriconazole and 48% in those treated with a lipid formulation of amphotericin B L-AMB: 10 mg/kg IV daily if CNS disease ABLC: not recommended in CNS disease One lipid formulation cannot be recommended over the other Use combination in critically ill patients pending on MIC values. De-escalate to monotherapy once MIC values are known IV voriconazole is recommended as initial therapy L-AMB: 10 mg/kg IV daily if CNS disease ABLC: not recommended in CNS disease If refractory to a lipid formulation of amphotericin B IV voriconazole is recommended as initial therapy with step-down to oral once clinically stable Options: Lipid formulation of amphotericin B ± terbinafine L-AMB/ABLC: 5 mg/kg IV daily L-AMB: 10 mg/kg IV daily if CNS disease ABLC: not recommended in CNS disease Terbinafine: 250 mg twice daily *Fusarium* spp. usually have high MIC values against posaconazole Need to know susceptibility results before use. As a result, it is only recommended as salvage therapy and only as the MR tablet or IV formulation L-AMB: 10 mg/kg IV daily if CNS disease ABLC: Not recommended in CNS disease Used if refractory to or intolerant of voriconazole	^56,95–97^ ^77^ ^96,98–101^ ^101^ ^101^ ^99,102–104^ ^77^

ABLC, amphotericin B lipid complex; c/w, compared with; EOT, end of therapy; L-AMB, liposomal amphotericin B; MR, modified-release; PO, oral; ROCM, rhino-orbital-cerebral mucormycosis; SEIFEM, Sorveglianza Epidemiologica Infezioni Fungine nelle Empoatie Maligne.

^a^Therapeutic drug monitoring recommended. Aim for a level of ≥1 mg/L for voriconazole, posaconazole and isavuconazole.^69–71,105^

^b^200 mg of isavuconazole is equal to 372 mg of isavuconazonium sulphate (prodrug of isavuconazole).

**Table 3.** Novel antifungal agents in development with potential to treat invasive fungal infection due to Mucorales, *Scedosporium* species, *Lomentospora prolificans* and *Fusarium* species

Antifungal agent	Mode of action	*In vitro* activity (*n*)^a^	*In vivo* activity^a^	Clinical trials	Relevant references
Fosmanogepix (formerly APX001)	GPI-anchor inhibitor	**Mucorales** *Cunninghamella bertholletiae* (10) MEC_90_ > 8^b^ *Lichtheimia* spp. (20) MEC_90_ > 8^b^ *Mucor circinelloides* (10) MEC_90_ ≤ 2–8^b^ *Rhizopus* spp. (30) MEC_90_ > 8^b^ ***Scedosporium* spp.** *S. apiospermum* (38) MEC_90_ 0.12–16^b^ *S. aurantiacum* (10) MEC_90_ 0.03^b^ *S. boydii* (10) MEC_90_ 0.12^b^ ***Lomentospora prolificans*** (38) MEC_90_ 0.06–0.12^b^ ***Fusarium* spp.** *F. oxysporum* (25) MEC_90_ 0.25–16^b^ *F. solani* (15) MEC_90_ 0.06^b^ *F. verticilloides* (10) MEC_90_ 16^b^	**Mucorales** Mouse model using strains with MEC values of 0.25 and 4.0 Fungal burden decreased by 1.3 and 1.97 log_10_ Ce/g lung tissue when dosed with 78 mg/kg (plus ABT) and 104 mg/kg (plus ABT) of FMGX, respectively. Potentially treatable with FMGX if MEC values are low ***Scedosporium* spp.** Significant increase in median survival from 7 (placebo) to 13 and 11 days in mice treated with 78 mg/kg (plus ABT) daily and 104 mg/kg (plus ABT) daily of FMGX, respectively ***Fusarium* spp.** Significant increase in median survival from 7 (placebo) to 12 and 10 days in mice treated with 78 mg/kg (plus ABT) daily and 104 mg/kg (plus ABT) daily of FMGX, respectively	Phase II trial completed. Evaluating *Aspergillus* and rare moulds. No results reported (NCT04240886)	^109–113^ ^111,112,114^ ^111,112^ ^111,112,114^
Olorofim (formerly 901318)	Fungal dihydroorotate dehydrogenase inhibitor	**Mucorales** No activity due to phylogenetic differences in the DHODH drug target ***Scedosporium* spp.** *S. apiospermum* (30) MIC_90_ 0.25^b^ Range 0.03–0.5^b^ *S. aurantiacum* (20) MIC_90_ 1^b^ Range 0.06–1^b^ *S. boydii* (30) MIC_90_ 0.25^b^ Range 0.06–0.5^b^ ***Lomentospora prolificans*** (30) MIC_90_ 0.5.^b^ Range 0.06–0.5.^b^ ***Fusarium* spp.** *F. dimerum* (2) MIC_50_ >2^b^ Range 2 to >2^b^ *F. moniliforme* (1) MIC_50_ 0.03^b^ *F. oxysporum* (5) MIC_50_ 2^b^ Range 0.12 to >2^b^ *F. solani* (11) MIC_50_ >2^b^ Range 2 to >2^b^ *F. verticilloides* (1). MIC_90_ 0.5.^b^	Neutropenic mouse model treated with olorofim (15 mg/kg, q8h) Survival in mice infected with *S. apiospermum*, *P. boydii* and *L. prolificans* at Day 10 post-infection was 80%, 100% and 100%, respectively c/w 20% in the untreated controls Decreased fungal burden in kidneys Day 3 post-infection	Phase IIb trial: Evaluating treatment of *Aspergillus*, *Coccidioides* and rare mould infections in patients with limited or no other options Successful EORTC-MSGERC overall responses at Day 42 and Day 84 were 55% and 36% for *Scedosporium* (*n* = 11) and 53% and 53% for *L. prolificans* (*n* = 17) (NCT03583164)	^115,116^ ^117^
Ibrexafungerp (formerly SCY078/MK-3118)	Triterpenoid antifungal. Inhibits 1,3-β-D-glucan synthase	**Mucorales** No activity ***Scedosporium* spp.** *S. apiospermum*/*P. boydii* (19) MEC_90_ 4^b^ Range 1–8^b^ ***Lomentospora prolificans*** (5) MEC_90_ 4^b^ Range 1–4^b^ ***Fusarium* spp.** No activity	Immunosuppressed mouse model infected with *Rhizopus delemar* (mucormycosis). Treated with ibrexafungerp (30 mg/kg PO twice daily) Survival to Day 21 significantly better in mice treated with ibrexafungerp c/w placebo (*P* < 0.002) and was equivalent to other antifungal agents. Treatment with ibrexafungerp and L-AMB resulted in significantly improved survival c/w monotherapy (*P* < 0.04). No *in vitro* activity (MEC >8) but has *in vivo* activity	Phase II trial (FURI) Evaluating the treatment of patients with IFI who are intolerant of or refractory to other standard antifungal agents Single arm, non-comparator and open-label with the primary outcome assessment of global response up to Day 180 of treatment with ibrexafungerp Trial is ongoing (NCT03059992)	^118,119^ ^118^ ^118^ ^118^
Oteseconazole (formerly VT-1161)	Tetrazole that selectively inhibits fungal CYP51	**Mucorales** *Rhizopus arrhizus* var. *arrhizus* (7) MIC range 0.25–2^b^ *R. arrhizus* var. *delemar* (5) MIC range 8 to >32	**Mucorales** Immunosuppressed mouse model of pulmonary infection with *R. arrhizus* var. *arrhizus* (MIC 1). Median survivals for mice treated with placebo, high-dose L-AMB, VT-116 7.5 mg/kg and VT-115 15 mg/kg were 5, 8, 8 and 9 days, respectively		^120^
VT-1598	Selectively inhibits fungal CYP51	**Mucorales** *R. arrhizus* (11) GM MIC 3.53^b^ Range 0.5 to >16^bc^			^121^

ABT, 1-Aminobenzotriazole; Ce, conidial equivalents; c/w, compared with; CYP51, cytochrome P51; DHODH, dihydroorotate dehydrogenase; EORTC-MSGERC, European Organization for Research and Treatment of Cancer—Mycoses Study Group Education and Research Consortium; FMGX, fosmanogepix; GM, galactomannan; GPI, glycosylphosphatidylinositol; IFI, invasive fungal infection; L-AMB, liposomal amphotericin B; MEC, minimum effective concentration; MEC_90_, 90% minimum effective concentration; MIC_50_, MIC required to inhibit the growth of 50% of isolates; MIC_90,_ MIC required to inhibit the growth of 90% of isolates; PO, oral.

^a^Values are mg/L.

^b^Tested using CLSI methodology.

^c^Four isolates had MIC >16 mg/L and three of the four isolates were identified as *R. arrhizus* var. *delemar* by internal transcriber sequence (ITS) sequencing.

Surgical debridement, where feasible, is strongly recommended as survival rates are higher in those who have surgery with complete resection as compared with those who do not (Table 4).^6,8–10,28,124–126^ Early consultation with surgical colleagues and a multidisciplinary approach to the management of mucormycosis is key to optimizing survival. Repeated surgical debridement to ensure complete resection may be required in some cases. Adjunctive therapies such as hyperbaric oxygen, growth factors and granulocyte infusions can only be recommended on a case-by-case basis as the data are conflicting or limited to case series or single reports.^24,27,28,128–131^ It is also difficult to determine their true efficacy as they are often given in combination with antifungal therapy and surgery (Table 4). Novel adjunctive therapies include check-point inhibitors (e.g. nivolumab) with or without IFN-γ, antibodies against the peptides of CotH (protein kinases that play a role in morphogenesis, stress adaptation and virulence), and antibodies against integrin-β1, which hold promise for the future treatment of mucormycosis.^143–146^  *R. oryzae*-specific cytotoxic T cells have been shown to have *in vitro* activity, but use is limited to case reports (Table 4).^135^ More data are required before these can be recommended for widespread use in clinical practice.

**Table 4.** Adjunctive treatments for invasive fungal infection due to Mucorales, *Scedosporium* species, *Lomentospora prolificans* and *Fusarium* species

Organism	Adjunctive treatment	Comments	Relevant references
Mucorales	Surgical debridement	To completely resect devitalized tissue. Greatest level of evidence is for Mucorales (of all the NAMs). Multiple studies showing improved survival. Multiple surgical operations may be required	6,8–10,28,124–126
	Line removal	To remove an ongoing source of infection. Recommended to remove all indwelling catheters in patients with fungaemia, if feasible. Extrapolated from data on candidaemia	127
	Growth factors: G-CSF/GM-CSF	To augment host response. True efficacy is difficult to ascertain as given with other treatments. As a result, the data are conflicting, with one study showing no increase in survival [6/38 (21%) with G-CSF versus 2/9 (22%) in those who were not given G-CSF] and another reporting survival in 83% of cases of mucormycosis	24,28
	Granulocyte infusions	To use the infusions as a bridge until the patient’s own neutrophils recover. Given with other treatments so true efficacy is difficult to ascertain; 2/7 (29%) who received granulocyte transfusions survived in one retrospective study	27,28
	Hyperbaric oxygen	To inhibit fungal growth (*in vitro* models). Case reports/case series in patients with mucormycosis showing an association with improved survival: 4/7 (57.1%) who were not treated with hyperbaric oxygen died c/w 2/6 (33%) of those who were treated with hyperbaric oxygen in one retrospective study. Better outcomes for mucormycosis cases where DM is the underlying disease c/w haematological malignancy: 94% survival in those who had DM and got hyperbaric oxygen c/w 33% (*P* = 0.02) of those who had an underlying haematological malignancy or BMT and got hyperbaric oxygen. Biases are likely present	128–131
	DM	To treat the underlying risk. Hyperglycaemia promotes fungal proliferation and impairs phagocytosis and chemotaxis. DKA temporarily disrupts the ability of transferrin to bind iron, decreasing a key host defence, allowing Mucorales to proliferate. Reverse any DKA and achieve good control of DM	132–134
	Adaptive immunotherapy	To augment host immune response. Anti-*Rhizopus oryzae* T cells can be produced that increase the activity of host phagocytes. Cross-react with some but not all of the organisms of the Mucorales order	135
*Scedosporium* spp.	Surgical debridement	To completely resect devitalized tissue. No data specific to invasive infection due to *Scedosporium* spp. in terms of improved outcomes. Extrapolated from data on Mucorales and other NAMs. Multiple surgical operations may be required (93% of cases in one study)	136
	Line removal	To remove an ongoing source of infection. Recommended to remove all indwelling catheters in patients with fungaemia, if feasible. Extrapolated from data on candidaemia	
	Growth factors: G-CSF/GM-CSF	To augment host response. True efficacy is difficult to ascertain as given with other treatments. GM-CSF may augment PMN oxidative burst against *Scedosporium* spp.	
	Hyperbaric oxygen	To inhibit fungal growth (*in vitro* models). No data specific to invasive infection due to *Scedosporium* spp. Extrapolated from data on Mucorales	
	DM	To treat the underlying risk. No data specific to invasive infection due to *Scedosporium* spp. Extrapolated from data on Mucorales	
*Lomentospora prolificans*	Surgical debridement	To completely resect devitalized tissue. Associated with improved survival in cases of invasive infection due to *L. prolificans* (5% vs 30%; *P* = 0.045). Multiple surgical operations may be required	91
	Line removal	To remove an ongoing source of infection. Recommended to remove all indwelling catheters in patients with fungaemia, if feasible. Extrapolated from data on candidaemia	
	Growth factors: G-CSF/GM-CSF	To augment host response. G-CSF in combination with L-AMB was associated with improved survival c/w L-AMB monotherapy in a murine model (9.1 vs 13.2 days). GM-CSF in combination with posaconazole decreased the burden of infection in some organs [brain: 3.41 ± 0.10 (control) vs 3.23 ± 0.14 (POS-GM-CSF) log cfu/g; *P* < 0.05) but did not improve survival [7.0 ± 0.35 (controls) vs 7.3 ± 0.33 days (POS-GM-CSF); *P* = NS] in a murine model. G-CSF use in human survivors of invasive infection due to *L. prolificans* has been described. In one study of 109 patients, 20 (18.3%) were given G-CSF and 6 survived (30%). A case series from France reported that all five neutropenic patients given G-CSF survived	45,82,137–139
	Hyperbaric oxygen	To inhibit fungal growth (*in vitro* models). No data specific to invasive infection due to *L. prolificans*. Extrapolated from data on Mucorales	
	DM	To treat the underlying risk. No data specific to invasive infection due to *L. prolificans*. Extrapolated from data on Mucorales	
*Fusarium* spp.	Surgical debridement	To completely resect devitalized tissue. Associated with improved survival in cases of invasive infection due to *Fusarium* spp.: 5/6 (83.3%) patients who had skin lesions resected survived Multiple surgical operations may be required. Cases of endophthalmitis need to be co-managed with an experienced ophthalmologist	100,140
	Line removal	To remove an ongoing source of infection. Recommended to remove all indwelling catheters in patients with fungaemia, if feasible. Extrapolated from data on candidaemia	
	Growth factors: G-CSF/GM-CSF	To augment host response. Patients administered G-CSF had a response rate of 41%	141
	Granulocyte transfusion.	To use the infusions as a bridge until the patient’s own neutrophils recover: 10/11 (91%) patients treated with granulocyte transfusions in addition to antifungals had a favourable response	142
	Hyperbaric oxygen	To inhibit fungal growth (*in vitro* models). Extrapolated from data on Mucorales	
	DM	To treat the underlying risk. No data specific to invasive infection due to *Fusarium* spp. Extrapolated from data on Mucorales	

BMT, bone marrow transplant; c/w, compared with; DKA, diabetic ketoacidosis; DM, diabetes mellitus; G-CSF, granulocyte colony-stimulating factor; L-AMB, liposomal amphotericin B; NAM, non-*Aspergillus* moulds; NS, non-significant; PMN, polymorphonuclear; POS, posaconazole.

## *Scedosporium* species

Although ubiquitous, these saprophytic hyaline moulds are mostly found in temperate climates (e.g. Australia) resulting in geographical variations in incidence.^46,147,148^ The most common species include *S. apiospermum* species complex (SC), *S. aurantiacum* and *S. boydii* (formerly *Pseudallescheria boydii*).^46,149^

### Risk factors

Until recently, *Lomentospora prolificans* (formerly *S. prolificans*) was classified with other *Scedosporium* spp.^149^ Thus, much of the epidemiological data are intertwined, making it difficult to tease out specific risk factors for *Scedosporium* spp. vis-à-vis *L. prolificans*.

Severe neutropenia and T cell immunodeficiency have been identified as risk factors, and scedosporiosis is found more commonly in patients with a haematological malignancy or post-allogeneic HSCT, especially those with graft-versus-host disease (GVHD).^93^

### Diagnosis

**Figure 3.**
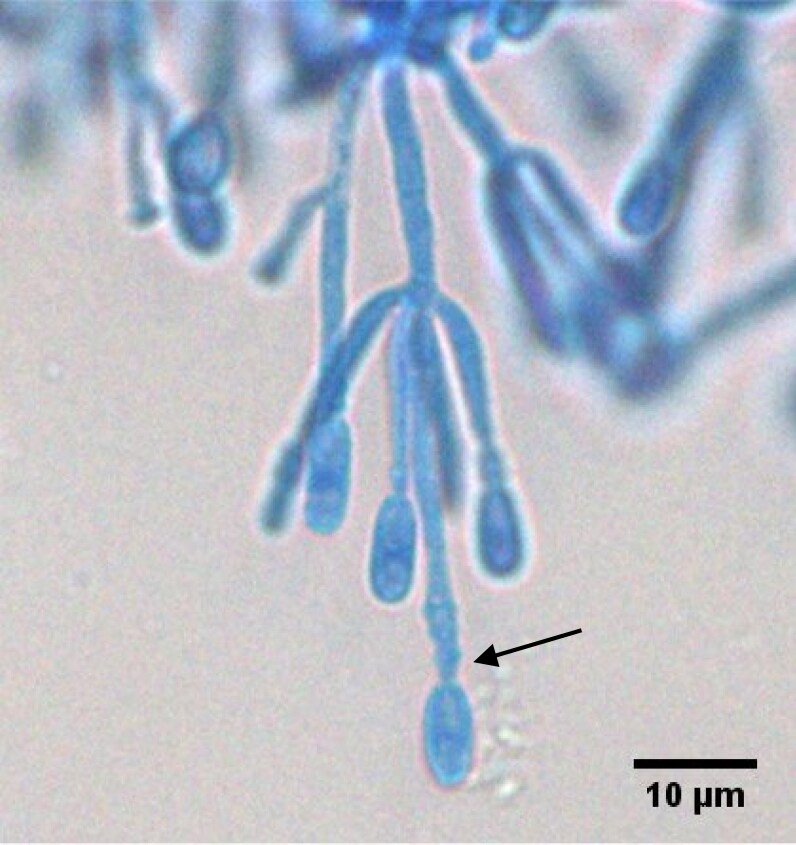
Single-forming anelloconidia of *Scedosporium apiospermum* attached to conidiophore, showing anellations (arrow) leading to the truncated base of the anelloconidia. Stained using lactophenol blue under coverslip. Courtesy of Professor Wieland Meyer, Westerdijk Fungal Biodiversity Institute and Ms Krystyna Maszewska, Dr Catriona Halliday, Mr Alex Khan, Mr Tsung-Yu Pai and Ms Georgia Clementine Wunderlich, Centre for Infectious Diseases and Microbiology, Westmead Hospital, Westmead, NSW, Australia.

**Figure 4.**
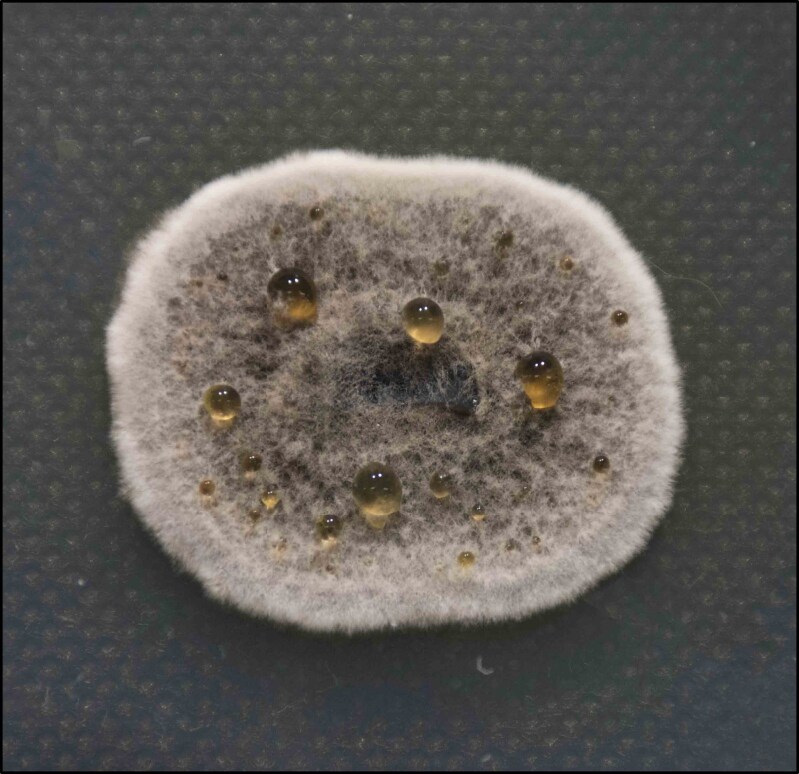
Top of *Scedosporium apiospermum* colony after 4 days of growth at 27°C on Sabouraud dextrose agar. Courtesy of Professor Wieland Meyer, Westerdijk Fungal Biodiversity Institute and Ms Krystyna Maszewska, Dr Catriona Halliday, Mr Alex Khan, Mr Tsung-Yu Pai and Ms Georgia Clementine Wunderlich, Centre for Infectious Diseases and Microbiology, Westmead Hospital, Westmead, NSW, Australia.

As *Scedosporium* spp. commonly disseminate, blood cultures and a detailed examination of the skin with biopsy of any suspicious lesion should be performed (Table 1). Like other NAMs, imaging is performed to determine the sites and extent of infection, detect any suitable sites for biopsy and assess responses to treatment. There are no characteristic imaging lesions that distinguish scedosporiosis from other NAM infections (Table 1). Microscopy, culture, histology and molecular tests are central to the accurate and timely diagnosis of scedosporiosis (Table 1 and Figures 3 and 4). Culture is generally required to identify the isolate to species level and for susceptibility testing (Table 1). Currently available molecular assays are not standardized or species-specific and have only been studied in the cystic fibrosis (CF) population (Table 1).^40^ Pan-fungal PCR assays can be used on tissue specimens (sensitivity of 94.4%) and ITS1, ITS2 and β-tubulin and calmodulin sequencing of cultures are recommended as an adjunct for diagnosis, if available (Table 1).^40–42^ GM assays do not detect *Scedosporium* spp. so are not recommended for use in diagnosis (Table 1). However, a meta-analysis demonstrated that 1,3-β-D-glucan assays have a sensitivity of 80.0% for the detection of invasive scedosporiosis in serum. Sixteen cases of proven or probable invasive scedosporiosis were included in the meta-analysis.^39^ As such, 1,3-β-D-glucan testing can be recommended as part of the diagnostic work-up for invasive scedosporiosis.

### Treatment

Again, a multi-pronged approach to treatment is required to optimize outcomes. First-line antifungal treatment is with voriconazole (IV; 6 mg/kg 12-hourly for two doses then 4 mg/kg 12-hourly thereafter) (Table 2).^82,83^ Recommended second-line treatment is with posaconazole (modified-release tablets/IV) with/without terbinafine (depending on the MIC value of posaconazole) (Table 2).^46,83–87^ An echinocandin or GM-CSF can be added to prior voriconazole monotherapy for combination salvage therapy with reported successful outcomes (Table 2).^89^ Once a patient is clinically stable or a partial response is detected then step-down from IV to oral therapy is recommended. Table 2 gives a detailed description of all the recommended antifungal regimens.

Novel antifungal agents that appear to have efficacy for scedosporiosis include fosmanogepix and olorofim (Table 3). MEC values for fosmanogepix against *Scedosporium* spp. are as low as MEC_90_ 0.03 mg/L (Table 3).^111,112^ Olorofim inhibits fungal dihydroorotate dehydrogenase, consequently inhibiting pyrimidine and DNA synthesis. Low MIC values against *Scedosporium* spp. have been detected by susceptibility testing (Table 3).^115^ A Phase IIb single-arm study of olorofim (NCT03583164) in the treatment of 202 patients with proven invasive *Aspergillus* or NAM infections intolerant of, resistant to or refractory to commercially available antifungal agents showed a complete or partial response of 36.4% (8/22) at Day 42%, and 22.7% (5/22) at Day 84 for infections with *Scedosporium* spp.^150^ Ibrexafungerp (a semi-synthetic derivative of enfumafungin that inhibits 1,3-β-D-glucan biosynthesis by a different pathway to echinocandins) has detected MIC values of 1–8 mg/L against *Scedosporium* spp. (Table 3).^118^

Surgical debridement, particularly of localized lesions in skin, bones and joints, is recommended (Table 4). One study reported that in 93% of cases multiple debridement was required.^148^ In all cases of fungaemia, it is strongly recommended that IV lines are removed (if feasible) (Table 4). This recommendation is extrapolated from the evidence on candidaemia. GM-CSFs augment human leucocyte activity against *Scedosporium* spp. (Table 4).^136^ There are no data on the use of hyperbaric oxygen to treat invasive scedosporiosis.

## Lomentospora prolificans

*L. prolificans* is a dematiaceous hyphomycete that has recently been determined to be phylogenetically distinct from *Scedosporium* spp.^149,151^ It is found in the soil of hot and dry climates, so its incidence is higher in Australia, Spain and the USA.^46,148,152^

### Risk factors

Risk factors include a haematological malignancy and severe neutropenia.^148^ Recipients of an HSCT are more likely than SOT patients to have invasive infection (39% versus 17%; *P* = 0.045) and for it to be fungaemic/disseminated.^82^ Breakthrough infections on voriconazole prophylaxis have also been identified, but are less common than for Mucorales.^45,153,154^

### Diagnosis

**Figure 5.**
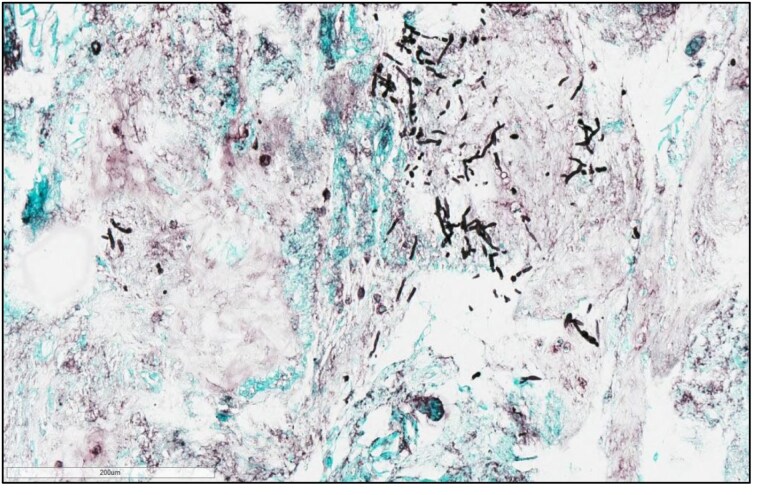
Histological specimen showing pigmented hyphae of *Lomentospora prolificans*. Grocott–Gömöri methenamine silver stain. ×100 magnification. Courtesy of Professor Catriona McLean, Anatomical Pathology, Alfred Health, Melbourne, Australia.

As *L. prolificans* commonly disseminates, blood cultures and a detailed examination of the skin with biopsy of any suspicious lesion should be performed (Table 1). Like other NAMs, imaging is critical to determine the sites and extent of infection, detect any suitable sites for biopsy and assess responses to treatment (Table 1). There are no characteristic imaging lesions that distinguish lomentosporiosis from other NAM infections. Specimens for microscopy, culture, histology and molecular testing are critical to early and accurate diagnosis (Table 1). On direct microscopy *L. prolificans* has pigmented hyphae (Figure 5). *L. prolificans* can be differentiated from *Scedosporium* spp. as it does not grow in cycloheximide.^149^ Several molecular assays are available (Table 1).^41,47–52^ None of these are standardized and have only been examined in CF patients. Until standardization occurs, the recommended format is a broad based (e.g. pan-fungal) assay with subsequent sequencing, hybridization or microarray (Table 1).^41,46–51,53^ Serological testing with GM is not recommended as it cannot be detected in the cell wall of *L. prolificans* (Table 1). Ten cases of proven or probable invasive fungal infection (IFI) due to *L. prolificans* were examined using 1,3-β-D-glucan assays in serum, demonstrating a sensitivity of 81.2%.^39^ Thus, 1,3-β-D-glucan testing can be recommended as part of the diagnostic work-up for IFI due to *L. prolificans*.

### Treatment

A multi-pronged approach to treatment is also recommended for invasive infection due to *L. prolificans*. First-line antifungal treatment is with voriconazole and terbinafine (Table 2).^83–87,91,92^ Second-line therapy is voriconazole-based (e.g. voriconazole plus L-AMB, voriconazole plus micafungin) (Table 2).^83,87,91^ Table 2 gives a detailed description of all the recommended antifungal regimens. Olorofim is the first antifungal agent that has been shown to be effective against *L. prolificans* with low median MIC values detected (Table 3).^115^ The Phase IIb single-arm study of olorofim (NCT03583164) showed a complete or partial response of 42.3% at Days 42 and 84, respectively, for *L. prolificans* invasive infections.^150^ Fosmanogepix also has activity against *L. prolificans*, with an MEC_90_ of 0.06–0.12 mg/L. MIC values for ibrexafungerp are higher at 1–4 mg/L (Table 3).^111,112^

Surgical debridement has been associated with an improved survival (30% versus 5%; *P *= 0.045).^91^ it is particularly effective in the setting of localized lesions of the skin, bone or joints (Table 4). Lines should be removed in the setting of fungaemia (if feasible). In a murine model of disseminated infection, the addition of granulocyte (G)-CSF to amphotericin B was associated with improved survival (Table 4).^137^ Survival of cases where G-CSF has been used has been reported.^45,82,138^ The addition of GM-CSF to posaconazole reduced the burden of infection in some organs in a murine model of disseminated infection but had no impact on survival.^139^ As such, where available, G-CSF/GM-CSF can be recommended as adjunct therapy for the treatment of *L. prolificans* infection, particularly in those cases of prolonged neutropenia (Table 4). There are no data on the use of granulocyte transfusions in patients with invasive lomentosporiosis; however, extrapolating from other NAM infections they can be considered in neutropenic patients whilst awaiting recovery of their own neutrophils (Table 4).

## *Fusarium* species

*Fusarium* spp. are hyaline moulds that are found mainly in soil. There are over 300 phylogenetically distinct species, which make up approximately 20 SCs.^155^ Ten SCs are associated with human infection, with the most common being *F. solani* SC (40%–60%), *F. oxysporum* SC (approximately 20%), *F. fujikuroi* SC (10%) and *F. moniliforme* SC (10%).^156^ Overall, *Fusarium* is the second most common NAM; however, it predominates in tropical and subtropical areas such as Brazil (13.1/1000 patients with acute myeloid leukaemia).^157,158^

### Risk factors

Risk factors include haematological malignancy, particularly those with advanced disease and poor performance status.^159^ Allogeneic HSCT recipients are at risk too, especially those with T cell impairment (e.g. with GVHD).^160^ Dissemination is common as the fungus can undergo adventitious sporulation in tissue and blood and it is intrinsically resistant to many antifungal agents, contributing to the high mortality rates seen.^95^

### Clinical manifestations

Paronychia is common with this fungus, so in addition to performing blood cultures and a skin examination, the nails need to be closely examined (Table 1).^161^ This may identify additional sites for swabbing and biopsy. Endophthalmitis can occur in the setting of dissemination, so in cases where fusariosis is suspected an ophthalmoscopic examination should be performed (Table 1). If endophthalmitis is detected then the appropriate ophthalmic specimens should be collected for microscopy, culture, histological examination and molecular testing. Consultation with an ophthalmologist is critical.

### Diagnosis

**Figure 6.**
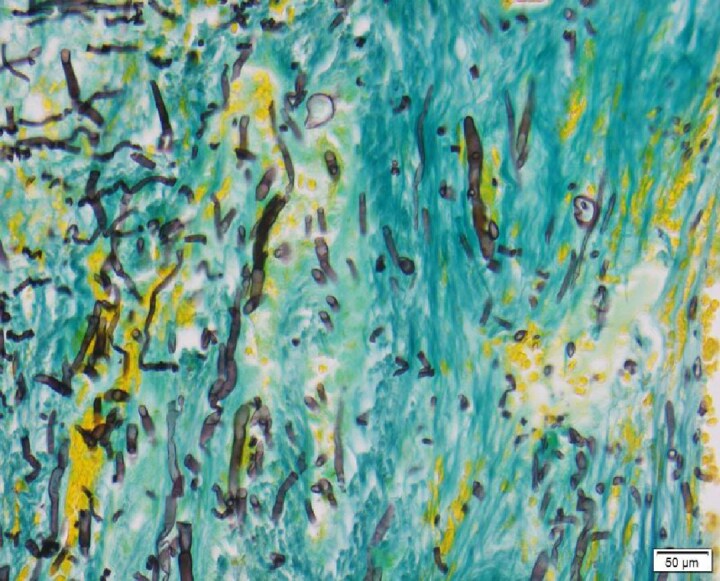
Histological specimen showing pigmented hyphae of *Fusarium*. Grocott–Gömöri methenamine silver stain. ×100 magnification. Courtesy of Professor Catriona McLean, Anatomical Pathology, Alfred Health, Melbourne, Australia.

**Figure 7.**
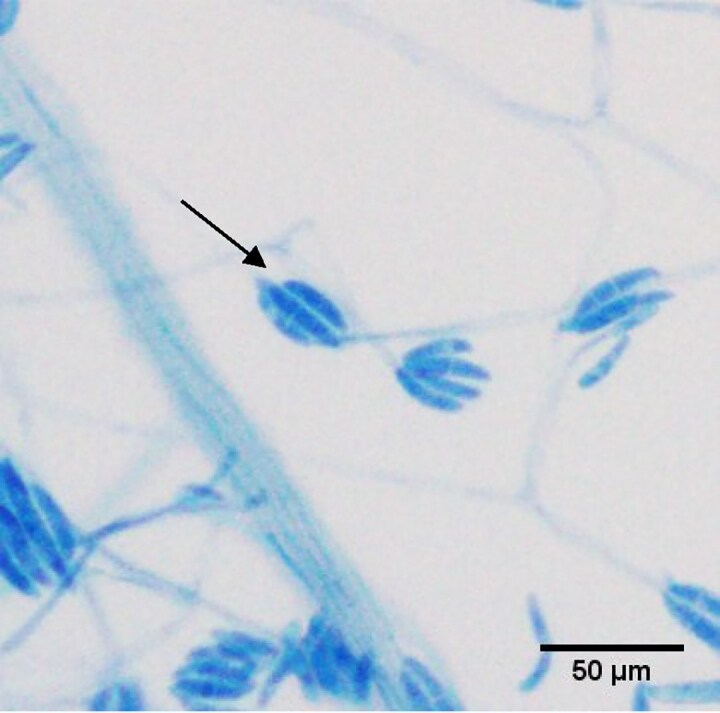
Banana- or canoe-shaped macroconidia of *Fusarium solani* can be observed forming on a short phialide/conidiophore (arrow). Prepared using adhesive tape method and stained using lactophenol blue. Courtesy of Professor Wieland Meyer, Ms Krystyna Maszewska, Dr Catriona Halliday, Mr Alex Khan, Mr Tsung-Yu Pai and Ms Georgia Clementine Wunderlich, Westerdijk Fungal Biodiversity Institute and Centre for Infectious Diseases and Microbiology, Westmead Hospital, Westmead, NSW, Australia.

CT scans of the chest are less likely to show halo-sign(s) than in invasive aspergillosis (Table 1).^55,56^ Other imaging should be performed to determine the sites and extent of infection, detect any suitable sites for biopsy and assess responses to treatment. Culture should be performed for full species identification.^162^ If *Fusarium* is suspected, then histology specimens should be stained with periodic acid–Schiff and Grocott–Gömöri methenamine stains as they assist in the identification of the hyphae of *Fusarium* spp. (Figure 6). Microscopically the hyaline hyphae with pigmented banana- or canoe-shaped multicellular macroconidia with a foot cell at the base are characteristic (Figure 7). Adventitious sporulation can be seen on microscopy, which differentiates *Fusarium* from *Aspergillus* spp. Several molecular methods are available.^163–166^ Sequencing of the *TEF1-α* gene can differentiate between the different species, and when combined with multiplex PCR or DNA microarray hybridization it can accurately identify *F. solani* and *F. oxysporum* in neutropenic patients (Table 1).^48,51,60,61^ The combination of *TEF1-α* with pan-fungal (ITS) PCR creates a pan-*Fusarium* assay that can be used in a variety of tissues including blood and CSF (Table 1).^41,50,51,62,63^ Species identification is very important as antifungal susceptibility is species dependent.^65^ The second largest subunit (RPB2) of RNA polymerase II has specific utility for species identification.^64^ Serological assays have utility in the diagnosis of fusariosis (Table 1). *Aspergillus* GM assay cross-reacts with *Fusarium* with a sensitivity and specificity of 83% and 67%, respectively.^58^ In most cases (73%) it detects infection before signs and symptoms are present.^58^ 1,3-β-D-glucan assay has a sensitivity and specificity of 90% and 60%, respectively.^59^ If readily available (short turnaround time) then these serological tests can be used as an adjunct in the diagnosis of invasive fusariosis (Table 1).

### Treatment

Treatment of infections due to *Fusarium* spp. is challenging due to their intrinsic resistance to many antifungal agents. Either voriconazole or L-AMB/amphotericin B lipid complex (5 mg/kg usually) are preferred as first-line treatment of fusariosis (Table 2).^56,95–97^ There are insufficient data to recommend one over the other. If a patient has refractory disease or is intolerant to one of voriconazole or L-AMB, then switching to the other antifungal agent is recommended (Table 2). If the patient is critically ill with fusariosis, then L-AMB and voriconazole can be used in combination until MIC results are available, then antifungal treatment can be rationalized (Table 2).^96,98–101^  *Fusarium* spp. generally have high MIC values to posaconazole. Posaconazole use should be guided by susceptibility testing and only used as salvage therapy, as modified-released tablets or IV formulations and with therapeutic drug monitoring.^99,102–104^ Table 2 gives a detailed description of all the recommended antifungal regimens. Fosmanogepix has *in vitro* activity against *F. solani* and *F. oxysporum* (MEC_90_ 0.06 and 0.25–16 mg/L, respectively) (Table 3).^112^ Fosmanogepix was successfully used to treat patients who were part of the recent Mexican outbreak of *Fusarium* meningitis.^167^ Olorofim has variable activity against *Fusarium* spp. In the Phase II study (NCT03583164) olorofim could only be used if *Fusarium* spp. had low MIC values (Table 3).

Successful outcomes have been reported with surgery in cases of fusariosis involving the skin, bones and joints (Table 4).^100,140^ In cases with endophthalmitis, the involvement of an experienced ophthalmologist is strongly recommended. Intravitreal instillation of antifungal agents and vitrectomy may be required. Since voriconazole has good penetration into the eye we recommend this agent as first-line for intravitreal injections (Table 4). Like other NAMs, lines should be removed in the setting of fungaemia (if feasible). The use of G-CSF and granulocyte transfusions as adjuncts in the treatment of invasive fusariosis should be considered as they have been associated with a favourable outcomes of 41% and 91% (*n* = 11), respectively (Table 4).^141,142^ There are no data on the use of hyperbaric oxygen in cases of invasive fusariosis.

## Other rare moulds

There are many other NAMs that rarely cause infection and if so it is mainly in the immunocompromised. A summary of the risk factors, diagnosis and treatment of these can be found in Table S1 (includes the related references) (available as Supplementary data at *JAC* Online). A more detailed description is beyond the scope of this review, but an excellent overview can be found in the global guidelines authored by Hoenigl *et al*.^168^

## Conclusions

NAM infections are on the increase and are associated with high mortality rates. Hence, early diagnosis is critical to improve outcomes. To achieve this, clinicians need to have a high level of suspicion and a knowledge of the risk factors so that prompt investigation can be performed. Identification to species level is critical to guide antifungal therapy. Microscopy, culture and histology were the cornerstone of diagnosis, but molecular methods are becoming more important as new assays that can determine the fungus to species level are being developed. New antifungal therapies are being developed that have the potential to improve outcomes due to greater efficacy and less toxicity. Multidisciplinary care remains critical to optimizing outcomes. Ongoing improvements in the outcomes require a better understanding of the epidemiology and risk factors, the development of novel diagnostic tools and effective antifungal treatments, and a greater insight into the efficacy of the various adjunctive therapies.

## Supplementary Material

dkaf005_Supplementary_Data

## Supplementary data

Table S1 is available as Supplementary data at *JAC* Online.
