# Supplementary material for: Diagnosis and management of invasive fungal infections due to non-Aspergillus moulds
Source: J Antimicrob Chemother. 2025 Mar 14;80(Suppl 1):i17–39. doi: 10.1093/jac/dkaf005 (PMC11908538; doi:10.1093/jac/dkaf005)
Supplement: dkaf005_Supplementary_Data [file dkaf005_supplementary_data.docx]

**Table S1. Epidemiology, diagnosis, and management of invasive infection due to rare mold infection**.

| **Organism^$^** | **Microbiology** | **Clinical manifestations** | **Risk factors** | **Diagnosis** | **Treatment*** | **Relevant references** |
| --- | --- | --- | --- | --- | --- | --- |
| Non-*marneffei Talaromyces* spp. | Belong to the order Eurotiales.  Ubiquitous in the environment. | Lung involvement most common.  Dissemination is rare. | CGD. Malignancy.  Long-term corticosteroids. | Chest CT scan.  Microscopy.  Culture.  Histology:   - To show invasion with septate hyphae.   Molecular:   - Critical as morphological identification is difficult. - ITS, β-tubulin sequencing.   Susceptibility testing:   - To guide antifungal therapy. - Azole MIC values generally higher than echinocandins and amphotericin B MIC values. | First-line treatment:   - L-AMB^#^   Salvage therapy:   - Echinocandin plus terbinafine. - Voriconazole (dependent on MIC values).   Adjunctive therapy:   - Surgical debridement of infected tissue (lung nodules). | ^169-177^ |
| *Paecilomyces* *variotii*. | Hyaline mold.  Thermotolerant.  Global distribution. | Pneumonia.  Skin and soft tissue infections.  Osteomyelitis.  Peritonitis.  Fungaemia. | Breakthrough infection on voriconazole. | Blood cultures.  Chest CT scan:   - Ground-glass opacification or patchy consolidation.   Microscopy:   - Phialides are more tapered as c/w *Penicillium* spp.   Culture:   - Fast growing, powdery to suede-like, yellow/brown to sand colour, tufted.   Molecular:   - For species identification. - From cultures.   Susceptibility testing:   - To guide antifungal therapy. - Posaconazole and itraconazole has greatest activity. Voriconazole the least activity of the triazole. Terbinafine has activity. | First-line treatment:   - Lipid formulation of amphotericin B or posaconazole.   Salvage treatment:   - Posaconazole (if not used as first-line).   Combination therapy:   - Not routine, severe cases only. - L-AMB with itraconazole or anidulafungin.   Adjunctive therapy:   - Surgical debridement of infected tissue. - Removal of any implicated catheters. | ^178-185^ |
| *Penicillium* spp. | Ubiquitous in the environment.  Rarely invasive.  Commonly allergenic or a coloniser. | Opportunistic.  Keratitis.  Endophthalmitis.  Lungs.  Dissemination. |  | Chest CT scan.  Blood cultures.  Microscopy.  Culture:   - Deep tissue (as colonisation is common).   Histology:   - To show invasion (as colonisation is common).   Molecular:   - For species identification as morphological diagnosis is difficult. - ITS-, D1/D2- or 18S-based rDNA PCR assay should be used.   Serology:   - Cross reacts with *Aspergillus* GM and lateral flow assays. - BDG assay has also been used for diagnosis.   Antifungal susceptibility testing:   - Should be performed as the MIC values vary widely according to species. | Difficult as data are scarce.  Mainly case reports/case series with conflicting results.  First-line:   - L-AMB alone or in combination with other antifungals (especially for disseminated infection). - Alternate is posaconazole, for localised infection and where MIC for voriconazole is high.   Salvage:   - Voriconazole (dependent on MIC values).   Endophthalmitis   - May need intravitreal treatment. - Consult an experienced ophthalmologist.   Adjunctive therapy:   - Surgical debridement, as feasible. | ^74,154,169,175,177,186-199^ |
| *Purpureocillium* *lilacinum*. | Formerly *Paecilomyces lilacinus*.  Hyaline mold.  Ubiquitous in the environment. | Most commonly keratitis and endophthalmitis.  Cutaneous and subcutaneous infections in the ICH.  Healthcare outbreaks related to contaminated moisturising cream in haematology patients.  Catheter-related blood stream infection (Fungaemia) in cancer patients (n=2). | Ocular trophism.  Survives on polyurethane catheters.  Transplantation.  Haematological malignancy. | Blood cultures.  Microscopy:   - Adventitial sporulation.   Culture:   - Flat, powdery/velvety, pink, red or purple in colour.   Molecular:   - For species identification. - From cultures. - ITS sequencing.   Susceptibility testing:   - To guide antifungal therapy. - Resistant to amphotericin B. - Of the triazoles, posaconazole has the lowest MIC and voriconazole has very variable MIC values. | Frist-line treatment:   - Voriconazole (whilst waiting MIC values). - Voriconazole and terbinafine. - Posaconazole.   Salvage therapy:   - Posaconazole (if not used as first-line).   Adjunctive therapy:   - Surgical debridement of localised lesions, as feasible. - Removal of IV catheters in the setting of fungaemia. | ^182,200-210^ |
| *Rasamsonia* spp. | New genus (2011).  11 species.  Species previously classified under the genera -*Geosmithia*, *Penicillium,* or *Talaromyces.*  Thermotolerant. | Lung infections are most common and may extend to adjacent tissues.  Disseminated infection is rare.  Can colonise the airways of CF patients, sometimes with clinical significance. | Invasive infection in severely ill patients with CGD or underlying malignancy.  HSCT recipients.  Lung transplant recipients. | Blood cultures.  Imaging:   - Determine the extent of infection as it may spread to adjacent organs as well as disseminate.   Microscopy:   - Can be misidentified as *Paecilomyces* spp. or *Penicillium* spp. - Characterised by having regular branched conidiophores with rough walls and the conidial shape is cylindrical.   Culture:   - Pre-treat sputum with a mucolytic agent (disadvantage is that this can result in false positive GM results).   Histology:   - To show invasion (as colonisation can occur).   Serology   - GM assay cross reacts with *Rasamsonia* spp.   Molecular   - Important for species identification given difficulties with morphological differentiation. - ITS, β-tubulin sequencing. - Can be genotyped using repetitive sequence-based PCR and RAPD. - RT-PCR has been used on respiratory samples from CF patients.   Susceptibility testing:   - To guide antifungal therapy. - No interpretive breakpoints so high MIC values may not correlate with poor outcomes. | First-line   - Echinocandin or an echinocandin with L-AMB or posaconazole.   Avoid azole monotherapy as high MIC values have been reported and has been associated with clinical failure.  Adjunctive therapy:   - Surgical debridement, if feasible. | ^5,211-231^ |
| *Schizophyllum* and other basidiomycetes. | *S. commune,*  *Coprinopsis cinerea* (previously *Hormographiella aspergillata),* and *Phanerochaete*  *Chrysosporium* (previously *Sporotrichum pruinosum*) are the most common causes of human infection.  Found in decaying matter.  Global distribution. | Lungs and sinuses commonly affected.  Infections in the eye, brain, spine, and peritoneum have been reported, but are rare.  Fungaemia is uncommon. |  | Imaging.  Microscopy:   - Hyphal clamp connections with spicules characteristic of *S. commune*.   Culture:   - Sometime sterile as these organisms do not sporulate in the laboratory.   Histology:   - To show invasion.   Serology:  A positive GM usually indicates co-infection with *Aspergillus* as GM is not a component of the cell wall of basidiomycetes and false positive CRAG results can occur (can be misdiagnosed as cryptococcal infection as a result).  Molecular:   - ITS and/or D1/D2 sequencing.   Susceptibility testing:   - To guide antifungal therapy. - No interpretive breakpoints so high MIC values may not correlate with poor outcomes. | ***S. commune***  First-line therapy   - L-AMB with disseminated or CNS infection. Can step down to posaconazole once stable. - An azole (voriconazole) may be used if pulmonary disease alone is detected.   Salvage therapy:   - Voriconazole if intolerant of L-AMB.   ***C. cinerea***  First-line therapy:   - L-AMB (IV) ± inhaled L-AMB (if pulmonary disease) or voriconazole.   Salvage:   - Voriconazole IV if intolerant of L-AMB.   Avoid echinocandins as MIC values are high and clinical failure with their use has been reported. | ^132,232-254^ |
| *Scopulariopsis* spp. | Molecularly closely related to *Scedosporium* genus.  Global distribution.  Found in soil and plant matter.  *S. brevicaulis* most common. | In the ICH can cause lung, sinus, soft tissue and disseminated infection. |  | Culture:   - Needed for definitive diagnosis.   Molecular:   - Essential for differentiation between it and *Aspergillus* spp., *Fusarium* spp. and *Scedosporium* spp. - ITS-, D1/D2- or 18S-based rDNA PCR assay should be used for species identification.   Susceptibility testing:   - No breakpoints. - MIC values high for most antifungal agents (amphotericin B and itraconazole). - Should be performed on all clinical isolates to guide antifungal therapy. | No clear recommendation for first-line treatment.  *In vitro* synergy detected for the combinations of posaconazole and terbinafine, posaconazole and caspofungin, and amphotericin B and caspofungin.  L-AMB and voriconazole, voriconazole alone and lipid formulations of amphotericin B with other antifungal agents can be recommended.  Voriconazole with an echinocandin and terbinafine can also be recommended.  Adjunctive therapy:   - Surgical debridement (if feasible). - Reversal of host’s immunodeficiency (if feasible). | ^74,193,255-266^ |

CF, cystic fibrosis; CGD, chronic granulomatous disease; CNS, central nervous system; CRAG, cryptococcal antigen; CT, computed tomography; c/w, compared with; BDG, β-D-glucan; GM, galactomannan; HSCT, haemopoietic stem cells transplant; ICH, immunocompromised host; ITS, internal transcriber sequence; IV, intravenous; L-AMB, liposomal amphotericin B; MIC, minimum inhibitory concentration; PCR, polymerase chain reaction; RAPD, random amplification of polymorphic DNA; rDNA, ribosomal DNA; RT-PCR, real-time PCR; spp., species.

^$^Alphabetical order.

^*^For dosing of the antifungal agents, see Table 2.

^#^Most of the data is on L-AMB which has been successful as first-line treatment.

**References:**

169. Lyratzopoulos G, Ellis M, Nerringer R, *et al*. Invasive infection due to *Penicillium* species other than *P. marneffei*. *J Infect* 2002; **45**: 184-95.

170. Horré R, Gilges S, Breig P, *et al*. Case report. Fungaemia due to *Penicillium piceum*, a member of the *Penicillium marneffei* complex. *Mycoses* 2001; **44**: 502-4.

171. Breton P, Germaud P, Morin O, *et al*. Rare pulmonary mycoses in patients with hematologic diseases. *Rev Pneumol Clin* 1998; **54**: 253-7.

172. Santos PE, Piontelli E, Shea YR, *et al*. *Penicillium piceum* infection: diagnosis and successful treatment in chronic granulomatous disease. *Med Mycol* 2006; **44**: 749-53.

173. Villanueva-Lozano H, Treviño-Rangel RJ, Renpenning-Carrasco EW, *et al*. Successful treatment of *Talaromyces amestolkiae* pulmonary infection with voriconazole in an acute lymphoblastic leukemia patient. *J Infect Chemother* 2017; **23**: 400-2.

174. Sili U, Bilgin H, Masania R, *et al*. Successful treatment of an invasive fungal infection caused by *Talaromyces* sp. with voriconazole. *Med Mycol Case Rep* 2015; **8**: 21-3.

175. Reboux G, Rocchi S, Vacheyrou M, Millon L. Identifying indoor air *Penicillium* species: a challenge for allergic patients. *J Med Microbiol* 2019; **68**: 812-21.

176. Atalay A, Koc AN, Akyol G, *et al*. Pulmonary infection caused by *Talaromyces purpurogenus* in a patient with multiple myeloma. *Infez Med* 2016; **24**: 153-7.

177. Guevara-Suarez M, Sutton DA, Cano-Lira JF, *et al*. Identification and Antifungal Susceptibility of *Penicillium*-Like Fungi from Clinical Samples in the United States. *J Clin Microbiol* 2016; **54**: 2155-61.

178. Salle V, Lecuyer E, Chouaki T, *et al*. *Paecilomyces variotii* fungemia in a patient with multiple myeloma: case report and literature review. *J Infect* 2005; **51**: e93-5.

179. Steiner B, Aquino VR, Paz AA, *et al*. *Paecilomyces variotii* as an Emergent Pathogenic Agent of Pneumonia. *Case Rep Infect Dis* 2013; **2013**: 273848.

180. Feldman R, Cockerham L, Buchan BW, *et al*. Treatment of *Paecilomyces variotii* pneumonia with posaconazole: case report and literature review. *Mycoses* 2016; **59**: 746-50.

181. Houbraken J, Verweij PE, Rijs AJ, *et al*. Identification of *Paecilomyces variotii* in clinical samples and settings. *J Clin Microbiol* 2010; **48**: 2754-61.

182. Castelli MV, Alastruey-Izquierdo A, Cuesta I, *et al*. Susceptibility testing and molecular classification of *Paecilomyces* spp. *Antimicrob Agents Chemother* 2008; **52**: 2926-8.

183. Chamilos G, Kontoyiannis DP. Voriconazole-resistant disseminated *Paecilomyces variotii* infection in a neutropenic patient with leukaemia on voriconazole prophylaxis. *J Infect* 2005; **51**: e225-8.

184. Uzunoglu E, Sahin AM. *Paecilomyces variotii* peritonitis in a patient on continuous ambulatory peritoneal dialysis. *J Mycol Med* 2017; **27**: 277-80.

185. Bellanger AP, Cervoni JP, Faucher JF, *et al*. *Paecilomyces variotii* Fungemia in a Patient with Lymphoma Needing Liver Transplant. *Mycopathologia* 2017; **182**: 761-5.

186. Marvisi M, Balzarini L, Mancini C, *et al*. A new type of Hypersensitivity Pneumonitis: salami brusher's disease. *Monaldi Arch Chest Dis* 2012; **77**: 35-7.

187. Garg A, Stuart A, Fajgenbaum M, *et al*. Chronic postoperative fungal endophthalmitis caused by *Penicillium citrinum* after cataract surgery. *J Cataract Refract Surg* 2016; **42**: 1380-2.

188. Böhlke M, Souza PA, Menezes AM, *et al*. Peritonitis due to *Penicillium* and *Enterobacter* in a patient receiving continuous ambulatory peritoneal dialysis. *Braz J Infect Dis* 2007; **11**: 166-8.

189. DelRossi AJ, Morse D, Spagna PM, *et al*. Successful management of *Penicillium* endocarditis. *J Thorac Cardiovasc Surg* 1980; **80**: 945-7.

190. Swoboda-Kopec E, Wroblewska MM, Rokosz A, *et al*. Mixed bloodstream infection with Staphylococcus aureus and *Penicillium chrysogenum* in an immunocompromised patient: case report and review of the literature. *Clin Microbiol Infect* 2003; **9**: 1116-7.

191. Mok T, Koehler AP, Yu MY, E *et al*. Fatal *Penicillium citrinum* pneumonia with pericarditis in a patient with acute leukemia. *J Clin Microbiol* 1997; **35**: 2654-6.

192. Hesse SE, Luethy PM, Beigel JH, *et al*. *Penicillium citrinum*: Opportunistic pathogen or idle bystander? A case analysis with demonstration of galactomannan cross-reactivity. *Med Mycol Case Rep* 2017; **17**: 8-10.

193. Vollmer T, Störmer M, Kleesiek K, *et al*. Evaluation of novel broad-range real-time PCR assay for rapid detection of human pathogenic fungi in various clinical specimens. *J Clin Microbiol* 2008; **46**: 1919-26.

194. Odabasi Z, Mattiuzzi G, Estey E, *et al*. β- d -Glucan as a Diagnostic Adjunct for Invasive Fungal Infections: Validation, Cutoff Development, and Performance in Patients with Acute Myelogenous Leukemia and Myelodysplastic Syndrome. *Clinical Infectious Diseases* 2004; **39**: 199-205.

195. Swan SK, Wagner RA, Myers JP, *et al*. Mycotic endophthalmitis caused by *Penicillium* sp. after parenteral drug abuse. *Am J Ophthalmol* 1985; **100**: 408-10.

196. Kanda K, Takayama K, Enoki T, *et al*. Chronic postcataract endophthalmitis caused by *Penicillium* species in an immunocompetent patient. *Int Med Case Rep J* 2018; **11**: 259-62.

197. Chowdhary A, Kathuria S, Agarwal K, *et al*. Voriconazole-Resistant *Penicillium oxalicum*: An Emerging Pathogen in Immunocompromised Hosts. *Open Forum Infect Dis* 2014; **1**: ofu029.

198. Radulesco T, Varoquaux A, Ranque S, *et al.* A Case of Fungus Ball-Type Maxillary Sinusitis Due to *Penicillium Roqueforti*. *Mycopathologia* 2018; **183**: 439-43.

199. Chen M, Houbraken J, Pan W, *et al*. Pulmonary fungus ball caused by *Penicillium capsulatum* in a patient with type 2 diabetes: a case report. *BMC Infect Dis* 2013; **13**: 496.

200. Neely AN, Orloff MM. Survival of some medically important fungi on hospital fabrics and plastics. *J Clin Microbiol* 2001; **39**: 3360-1.

201. Pastor FJ, Guarro J. Clinical manifestations, treatment and outcome of *Paecilomyces lilacinus* infections. *Clin Microbiol Infect* 2006; **12**: 948-60.

202. Orth B, Frei R, Itin PH, *et al*. Outbreak of invasive mycoses caused by *Paecilomyces lilacinus* from a contaminated skin lotion. *Ann Intern Med* 1996; **125**: 799-806.

203. Tan TQ, Ogden AK, Tillman J, *et al*. *Paecilomyces lilacinus* catheter-related fungemia in an immunocompromised pediatric patient. J Clin Microbiol 1992;30:2479-83.

204. Chan-Tack KM, Thio CL, Miller NS, *et al*. *Paecilomyces lilacinus* fungemia in an adult bone marrow transplant recipient. *Med Mycol* 1999; **37**: 57-60.

205. Luangsa-Ard J, Houbraken J, van Doorn T, *et al*. *Purpureocillium*, a new genus for the medically important *Paecilomyces lilacinus*. *FEMS Microbiol Lett* 2011; **321**: 141-9.

206. Espinel-Ingroff A. *In vitro* fungicidal activities of voriconazole, itraconazole, and amphotericin B against opportunistic moniliaceous and dematiaceous fungi. *J Clin Microbiol* 2001; **39**: 954-8.

207. Ciecko SC, Scher R. Invasive fungal rhinitis caused by *Paecilomyces lilacinus* infection: Report of a case and a novel treatment. *Ear Nose Throat J* 2010; **89**: 594-5.

208. Turner LD, Conrad D. Retrospective case-series of *Paecilomyces lilacinu*s ocular mycoses in Queensland, Australia. *BMC Res Notes* 2015; **8**: 627.

209. Ezzedine K, Belin E, Guillet S, *et al*. Cutaneous hyphomycosis due to *Paecilomyces lilacinus*. *Acta Derm Venereol* 2012; **92:** 156-7.

210. Ono N, Sato K, Yokomise H, *et al*. Lung abscess caused by *Paecilomyces lilacinus*. *Respiration* 1999; **66**: 85-7.

211. Houbraken J, Spierenburg H, Frisvad JC. *Rasamsonia*, a new genus comprising thermotolerant and thermophilic *Talaromyces* and *Geosmithia* species. Antonie Van Leeuwenhoek 2012; **101**: 403-21.

212. Abdolrasouli A, Bercusson AC, Rhodes JL, *et al*. Airway persistence by the emerging multi-azole-resistant *Rasamsonia argillacea* complex in cystic fibrosis. *Mycoses* 2018; **61**: 665-73.

213. Babiker A, Gupta N, Gibas CFC, *et al*. *Rasamsonia* sp: An emerging infection amongst chronic granulomatous disease patients. A case of disseminated infection by a putatively novel *Rasamsonia argillacea* species complex involving the heart. *Med Mycol Case Rep* 2019; **24**: 54-7.

214. De Ravin SS, Challipalli M, Anderson V, *et al*. *Geosmithia argillacea*: an emerging cause of invasive mycosis in human chronic granulomatous disease. *Clin Infect Dis* 20110; **52**: e136-43.

215. Hong G, White M, Lechtzin N, *et al*. Fatal disseminated *Rasamsonia* infection in cystic fibrosis post-lung transplantation. *J Cyst Fibros* 2017; **16**: e3-e7.

216. Ishiwada N, Takeshita K, Yaguchi T, *et al*. The First Case of Invasive Mixed-Mold Infections Due to *Emericella nidulans* var. *echinulata* and *Rasamsonia piperina* in a Patient with Chronic Granulomatous Disease. *Mycopathologia* 2016; **181**: 305-9.

217. Machouart M, Garcia-Hermoso D, Rivier A, *et al*. Emergence of disseminated infections due to *Geosmithia argillacea* in patients with chronic granulomatous disease receiving long-term azole antifungal prophylaxis. *J Clin Microbiol* 2011; **49**: 1681-3.

218. Ocak I, Bollino G, Bering P, *et al*. *Rasamsonia argillacea* species complex myocarditis in a patient with chronic granulomatous disease. *Radiol Case Rep* 2019; **14**: 766-70.

219. Valentin T, Neumeister P, Pichler M, *et al*. Disseminated *Geosmithia argillacea* infection in a patient with gastrointestinal GvHD. *Bone Marrow Transplant* 2012; **47**: 734-6.

220. Giraud S, Favennec L, Bougnoux ME, *et al*. *Rasamsonia argillacea* species complex: taxonomy, pathogenesis and clinical relevance. *Future Microbiol* 2013; **8**: 967-78.

221. Doyon JB, Sutton DA, Theodore P, *et al*. *Rasamsonia argillacea* pulmonary and aortic graft infection in an immune-competent patient. *J Clin Microbiol* 2013; **51**: 719-22.

222. Giraud S, Pihet M, Razafimandimby B, *et al*. *Geosmithia argillacea*: an emerging pathogen in patients with cystic fibrosis. *J Clin Microbiol* 2010; **48**: 2381-6.

223. Masoud-Landgraf L, Badura A, Eber E, *et al*. Modified culture method detects a high diversity of fungal species in cystic fibrosis patients. *Med Mycol* 2014; **52**: 179-86.

224. Prattes J, Koidl C, Eigl S, *et al*. Bronchoalveolar lavage fluid sample pretreatment with Sputasol(®) significantly reduces galactomannan levels. J Infect 2015; **70**: 541-3.

225. Steinmann J, Giraud S, Schmidt D, *et al*. Validation of a novel real-time PCR for detecting *Rasamsonia argillacea* species complex in respiratory secretions from cystic fibrosis patients. N*ew Microbes New Infect* 2014; **2**: 72-8.

226. Ziesing S, Suerbaum S, Sedlacek L. Fungal epidemiology and diversity in cystic fibrosis patients over a 5-year period in a national reference center. *Med Mycol* 2016; **54**: 781-6.

227. Barker AP, Horan JL, Slechta ES, *et al*. Complexities associated with the molecular and proteomic identification of *Paecilomyces* species in the clinical mycology laboratory. *Med Mycol* 2014; **52**: 537-45.

228. Houbraken J, Giraud S, Meijer M, *et al*. Taxonomy and antifungal susceptibility of clinically important *Rasamsonia* species. *J Clin Microbiol* 2013; **51**: 22-30.

229. Mouhajir A, Matray O, Giraud S, *et al*. Long-Term *Rasamsonia argillacea* Complex Species Colonization Revealed by PCR Amplification of Repetitive DNA Sequences in Cystic Fibrosis Patients. *J Clin Microbiol* 2016; **54**: 2804-12.

230. Sohn JY, Jang MA, Lee JH, *et al*. Isolation and identification of *Geosmithia argillacea* from a fungal ball in the lung of a tuberculosis patient. *Ann Lab Med* 2013; **33**: 136-40.

231. Marguet C, Favennec L, Matray O, *et al.* Clinical and microbiological efficacy of micafungin on *Geosmithia argillacea* infection in a cystic fibrosis patient. *Med Mycol Case Rep* 2012; **1**: 79-81.

232. Michel J, Maubon D, Varoquaux DA, *et al*. *Schizophyllum commune*: an emergent or misdiagnosed fungal pathogen in rhinology? *Med Mycol* 2016; **54**: 301-9.

233. Pekic S, Arsenijevic VA, Gazibara MS, et al. What lurks in the sellar? *Lancet* 2010; **375**:432.

234. Hoenigl M, Aspeck E, Valentin T, *et al*. Sinusitis and frontal brain abscess in a diabetic patient caused by the basidiomycete *Schizophyllum commune*: case report and review of the literature. *Mycoses* 2013; **56**: 389-93.

235. Filipe R, Caldas JP, Soares N, *et al*. *Schizophyllum commune* sphenoidal sinusitis as presentation of a non-Hodgkin Lymphoma. *Med Mycol Case Rep* 2020; **28**: 26-8.

236. Conen A, Weisser M, Hohler D, *et al.* *Hormographiella aspergillata*: an emerging mould in acute leukaemia patients? *Clin Microbiol Infect* 2011; **17**: 273-7.

237. Godet C, Cateau E, Rammaert B, *et al*. Nebulized Liposomal Amphotericin B for Treatment of Pulmonary Infection Caused by *Hormographiella aspergillata*: Case Report and Literature Review. *Mycopathologia* 2017; **182**: 709-13.

238. Jain N, Jinagal J, Kaur H, *et al*. Ocular infection caused by *Hormographiella aspergillata*: A case report and review of literature. *J Mycol Med* 2019; **29**: 71-4.

239. Reddy AK, Ashok R, Majety M, *et al*. Fungal keratitis due to *Schizophyllum commune*: an emerging pathogenic fungus. *Mycoses* 2016; **59**: 757-9.

240. Chauhan A, Gruenberg J, Arbefeville S, *et al*. Disseminated *Hormographiella aspergillata* Infection with Lung and Brain Involvement after Allogenic Hematopoietic Stem-Cell Transplantation in a 54-Year-Old Man. *Lab Med* 2019; **50**: 426-31.

241. Tone K, Fujisaki R, Hagiwara S, *et al*. Epidural abscess caused by *Schizophyllum commune*: A rare case of rhinogenic cranial complication by a filamentous basidiomycete. *Mycoses* 2018; **61**: 213-7.

242. Oliveira MME, Lemos AS, Gonçalves MLC, *et al*. Fungemia associated with *Schizophyllum* *commune* in Brazil. *PLoS Negl Trop Dis* 2017; **11**: e0005549.

243. Chowdhary A, Randhawa HS, Gaur SN, *et a*l. *Schizophyllum commune* as an emerging fungal pathogen: a review and report of two cases. *Mycoses* 2013; **56:** 1-10.

244. Lagrou K, Massonet C, Theunissen K, *et al*. Fatal pulmonary infection in a leukaemic patient caused by *Hormographiella aspergillata*. J *Med Microbiol* 2005; **54**: 685-8.

245. Suarez F, Olivier G, Garcia-Hermoso D, *et al*. Breakthrough *Hormographiella aspergillata* infections arising in neutropenic patients treated empirically with caspofungin. J Clin Microbiol 2011; **49**: 461-5.

246. Haidar G, Zerbe CS, Cheng M, **et al**. *Phellinus* species: An emerging cause of refractory fungal infections in patients with X-linked chronic granulomatous disease. *Mycoses* 2017; **60**: 155-60.

247. Chan JF, Teng JL, Li IW, *et al*. Fatal empyema thoracis caused by *Schizophyllum commune* with cross-reactive cryptococcal antigenemia. *J Clin Microbiol* 2014; **52**: 683-7.

248. Surmont I, Van Aelst F, Verbanck J, *et al*. A pulmonary infection caused by *Coprinus cinereus* (*Hormographiella aspergillata*) diagnosed after a neutropenic episode. *Med Mycol* 2002; **40**: 217-9.

249. Verweij PE, van Kasteren M, van de Nes J, *et al*. Fatal pulmonary infection caused by the basidiomycete *Hormographiella aspergillata*. *J Clin Microbiol* 1997; **35:** 2675-8.

250. Williamson D, Pandey S, Taylor S, *et al*. A case of infection caused by the basidiomycete *Phellinus undulatus*. *J Med Microbiol* 2011; **60:** 256-8.

251. González GM, Sutton DA, Thompson E, *et al*. *In vitro* activities of approved and investigational antifungal agents against 44 clinical isolates of basidiomycetous fungi. A*ntimicrob Agents Chemother* 2001; **45**: 633-5.

252. Chowdhary A, Kathuria S, Singh PK, *et al*. Molecular characterization and *in vitro* antifungal susceptibility profile of *Schizophyllum commune*, an emerging basidiomycete in bronchopulmonary mycoses. *Antimicrob Agents Chemother* 2013; **57**: 2845-8.

253. Correa-Martinez C, Brentrup A, Hess K, *et al*. First description of a local *Coprinopsis cinerea* skin and soft tissue infection. *New Microbes New Infect* 2018; **21**: 102-4.

254. Tullio V, Mandras N, Banche G, *et al*. *Schizophyllum commune*: an unusual of agent bronchopneumonia in an immunocompromised patient. *Med Mycol* 2008; **46**: 735-8.

255. Sandoval-Denis M, Sutton DA, Fothergill AW, *et al*. *Scopulariopsis*, a poorly known opportunistic fungus: spectrum of species in clinical samples and *in vitro* responses to antifungal drugs. *J Clin Microbiol* 2013; **51**: 3937-43.

256. Iwen PC, Schutte SD, Florescu DF, *et al*. Invasive *Scopulariopsis brevicaulis* infection in an immunocompromised patient and review of prior cases caused by *Scopulariopsis* and *Microascus* species. *Med Mycol* 2012; **50**: 561-9.

257. Salmon A, Debourgogne A, Vasbien M, *et al*. Disseminated *Scopulariopsis brevicaulis* infection in an allogeneic stem cell recipient: case report and review of the literature. *Clin Microbiol Infect* 2010; **16:** 508-12.

258. Yang Q, Wei J, Chen Z. Fatal bronchial invasion of *Scopulariopsis brevicaulis* in an acute monocytic leukemia patient. *Diagn Microbiol Infect Dis* 2012; **73**: 369-71.

259. Aguilar C, Pujol I, Guarro J. *In vitro* antifungal susceptibilities of *Scopulariopsis* isolates. *Antimicrob Agents Chemother* 1999; **43**: 1520-2.

260. Cuenca-Estrella M, Gomez-Lopez A, Buitrago MJ, *et al*. *In vitro* activities of 10 combinations of antifungal agents against the multiresistant pathogen *Scopulariopsis brevicaulis*. *Antimicrob Agents Chemother* 2006; **50**: 2248-50.

261. Martin-Vicente A, Guarro J, Capilla J. Does a triple combination have better activity than double combinations against multiresistant fungi? Experimental *in vitro* evaluation. Int J Antimicrob Agents 2017; **49**: 422-6.

262. Kurata K, Nishimura S, Ichikawa H, *et al*. Invasive *Scopulariopsis alboflavescens* infection in patient with acute myeloid leukemia. *Int J Hematol* 2018; **108**: 658-64.

263. Perfect JR. Treatment of non-*Aspergillus* moulds in immunocompromised patients, with amphotericin B lipid complex. *Clin Infect Dis* 2005; **40** Suppl 6: S401-8.

264. Phillips P, Wood WS, Phillips G, *et al*. Invasive hyalohyphomycosis caused by *Scopulariopsis brevicaulis* in a patient undergoing allogeneic bone marrow transplant. Diagn Microbiol Infect Dis 1989; **12**: 429-32.

265. Miossec C, Morio F, Lepoivre T, *et a*l. Fatal invasive infection with fungemia due to *Microas*cus cirrosus after heart and lung transplantation in a patient with cystic fibrosis. *J Clin Microbiol* 2011; **49**: 2743-7.

266. Yao L, Wan Z, Li R, *et al*. *In Vitro* Triple Combination of Antifungal Drugs against Clinical *Scopulariopsis* and *Microascus* Species. Antimicrob Agents Chemother 2015; **59**: 5040-3.
